# Progressive Resistance Training in Parkinson’s Disease: An Umbrella Review Examining the Role of Methodological Adherence and Training Progression Principles in Clinical Outcome

**DOI:** 10.3390/jfmk11020178

**Published:** 2026-04-28

**Authors:** Ya’ara Rozenbaum, Yeshayahu Hutzler, Sharon Barak

**Affiliations:** 1 Lewinsky-Wingate Academic Center, Netanya 4290200, Israel; rozenbaumyaara@gmail.com; 2 Israel ParaSport Center, Ramat Gan 5253529, Israel; 3 Nursing Department, Faculty of Health Sciences, Ariel University, Ariel 40700, Israel; sharoni.baraki@gmail.com; 4 Department of Pediatric Rehabilitation, The Edmond and Lily Safra Children’s Hospital, The Chaim Sheba Medical Center, Ramat Gan 5266202, Israel

**Keywords:** Parkinson’s disease, progressive resistance training, strength, functional capacity, motor signs, umbrella review

## Abstract

**Objective:** The goal was to investigate the relationship between methodological adherence and clinical outcomes in Progressive Resistance Training (PRT) for Parkinson’s Disease (PD), specifically identifying why findings of “superiority” over active controls remain inconsistent. **Methods:** This umbrella review utilized a multi-stage process to identify a sample of the primary literature for methodological analysis. An initial search identified 38 systematic reviews published within the specified timeframe. From the reference lists of these reviews, a subset of 34 primary clinical studies was purposefully selected. Inclusion was prioritized for studies providing comprehensive methodological data on PRT protocols and standardized clinical outcomes. Interventions were evaluated using a three-tiered framework: (1) training protocol with specifications of Frequency, Intensity, Time, Type, Volume, and Progression (FITT-VP) (General Exercise), (2) FITT-VP integrated with the American College of Sports Medicine (ACSM) Supplementary Guidelines (Integrated Guidelines), and (3) principles of progression (mechanistic growth). Studies were categorized by control type (active (e.g., aerobic or balance), *n* = 26; passive (e.g., standard care or no exercise), *n* = 8). **Results:** In trials that compared PRT with an active control group, PRT achieved clinical superiority in 57% (*n* = 15) of trials and 46% (*n* = 12) when focusing on trials with an effect on specific functional or balance outcomes. Among these successful interventions, 75% maintained high adherence (≥70%) to the Integrated Guidelines, and 58% maintained high adherence to the principles of progression. In the 53% (*n* = 14) of studies where PRT was found non-superior (equivalent or inferior in functional or balance outcomes) to an active control, 0% met the high adherence threshold for progression. While general FITT-VP compliance remained high (78%), the failure to implement systematic load, specificity, and variation served as a definitive barrier to competitive superiority. In the 100% of studies where PRT outperformed passive controls, high progression was present in 57% of cases. This may suggest that while a baseline resistance stimulus outperforms inactivity, it is fundamentally insufficient to outperform other active clinical therapies. **Conclusions:** This umbrella review indicates that adherence to the principles of progression may be an important factor influencing the clinical outcomes of PRT in individuals with PD. The variability observed in the current literature suggests that inconsistent application of established exercise frameworks—rather than the failure of the modality itself—could be a contributing element to the reported “inconclusiveness.” To potentially enhance functional outcomes and the comparative effectiveness of PRT, future research should consider prioritizing structured adherence to FITT-VP, Integrated Guidelines, and progression-based frameworks. Establishing a 70% adherence threshold is proposed as a potential benchmark to improve protocol consistency and support rehabilitation efficacy in this population.

## 1. Introduction

### 1.1. Parkinson’s Disease

Parkinson’s Disease (PD) is a complex neurodegenerative disorder arising from an interplay of aging, environmental factors, and genetic susceptibility [1]. While the disease is predominantly characterized by the progressive loss of dopaminergic neurons within the substantia nigra, its clinical impact extends far beyond the central nervous system [2]. The incidence of PD rises with age and is currently escalating more rapidly than any other neurological condition, particularly among men [2]. The deterioration of neural pathways is compounded by age-related inactivity, which accelerates the loss of muscle mass and strength [1]. This intersection of neurological decay and skeletal muscle atrophy manifests as a constellation of motor symptoms, including tremors, bradykinesia, rigidity, and generalized muscular weakness [2]. Collectively, these impairments foster fear of falling, social isolation, and a significantly diminished quality of life (QoL) [3].

### 1.2. Exercise Training in the Rehabilitation of PD

Traditional management of PD relies heavily on pharmacological interventions, such as Levodopa and dopaminergic agonists. However, prolonged use often leads to severe side effects and diminishing efficacy over time [4]. While deep-brain stimulation offers a surgical alternative for medication-refractory motor fluctuations, it is reserved for advanced cases [5]. Consequently, physical exercise has emerged as a critical complementary therapy. There is a robust consensus that diverse modalities—including balance therapy, endurance [6], dance, functional training, mind–body interventions [7], and gait training [3]—provide substantial benefits for individuals with PD. Yet, despite the proliferation of research, a definitive hierarchy of exercise efficacy has not been established, leaving clinicians without an optimal protocol for superior outcomes [7].

Progressive Resistance Training (PRT) is uniquely suited to address the specific motor impairments of PD [1], as it targets muscular strength, power, hypertrophy, and coordination [8]. Research confirms that mechanical muscle function is severely impaired in PD patients, independent of medication effects, and that this loss of strength directly dictates the level of functional disability [9]. PRT not only enhances muscle and bone mass (ACSM’s Guidelines [10]) but also specifically mitigates bradykinesia [4]. Theoretically, PRT represents a preferable rehabilitative approach due to its multifaceted ability to address the integration of central nervous system dysfunction and skeletal muscle atrophy within a single modality [1]. To standardize these benefits, the American College of Sports Medicine (ACSM) prescribes the FITT-VP framework (Frequency, Intensity, Time, Type, Volume, and Progression) (FITT-VP) [10]. Recent evidence suggests that adherence to these guidelines correlates with improved motor function, mobility, and QoL [11]. However, a significant limitation exists in the current literature: many studies [11,12,13,14,15,16,17] fail to incorporate the Integrate Supplementary Clinical Guidelines (Integrated Guidelines), such as functional task specificity and individualized balance challenges, into their protocols [10]. Most critically, to avoid training plateaus and maximize “Muscular Fitness” [10], three core principles must be strictly applied: progressive overload, specificity, and variation (principles of progression). However, the true clinical impact of PRT remains difficult to verify due to inconsistencies in how these protocols are applied in research settings.

It seems there is a prevalent lack of distinction between merely performing “weighted exercises” and a true physiological PRT intervention. For a resistance training program to achieve continuous physical and performance improvements, simply engaging in the training is insufficient [8]; it requires the systematic application of specific training principles to drive neuromuscular adaptation. Furthermore, while numerous systematic reviews have attempted to summarize these findings, they often reach conflicting conclusions. This necessitates a high-level synthesis, such as an umbrella review, to evaluate the evidence base while rigorously accounting for the underlying training dosage and methodological adherence. This umbrella review aims to address this ambiguity by synthesizing the existing body of reviews. It hypothesizes that the perceived “inconclusiveness” of PRT’s superiority over other active therapies is not a failure of the modality itself, but rather a result of the insufficient implementation of established training principles—specifically regarding progressive overload, specificity, and variation.

### 1.3. Aims of the Review

The primary aim of this umbrella review is to synthesize and critically evaluate the PRT interventions in clinical studies extracted from existing systematic reviews and meta-analyses regarding their efficacy for individuals with PD.

The review assesses the methodological implementation of these interventions to establish whether an association exists between adherence to established training principles—specifically FITT-VP, Integrated Guidelines, and principles of progression frameworks—and the observed clinical outcomes. Specifically, this review seeks to do the following:

Descriptive Objectives:

1. To summarize the reported effects of PRT on motor symptoms, functional capacity, balance, and QoL in persons with PD, as reported across the existing systematic review and meta-analysis literature.
2. To critically analyze the variability in PRT protocols (e.g., intensity, volume, progression, specificity) implemented in the primary clinical studies underpinning these reviews.

Analytical Objectives:

1. To evaluate the adherence of PRT interventions in key clinical studies to the ACSM FITT-VP for persons with PD.
2. To appraise intervention adherence by integrating FITT-VP with Integrated Guidelines, identifying any significant discrepancies between standard and integrated evaluative frameworks.
3. To investigate the association between inconsistent adherence to established principles of progression (progressive overload, variation, and specificity) and the inconclusive findings regarding PRT’s superiority over other active interventions.

## 2. Materials and Methods

### 2.1. Formulation of the Focused Question and Protocol Registration

This umbrella review was conducted in accordance with the PRISMA 2020 guidelines. The methodology followed the principles for “Overviews of Reviews” as described in the Cochrane Handbook for Systematic Reviews of Interventions, ensuring a systematic approach to study selection, data extraction, and quality appraisal [18]. Using the Participants, Intervention(s), Comparator(s), and Outcome(s) (PICO) framework outlined in the Preferred Reporting Items for Systematic Reviews and Meta-Analysis (PRISMA) guidelines [19], a focused question was developed: “Is PRT is more efficient intervention compared to alternative physical training modalities on motor and non-motor outcomes in individuals with PD?” A protocol was created and registered on PROSPERO (CRD420251170469) before the initiation of this umbrella review. The complete PRISMA checklist is available as Table S1 [20].

### 2.2. Eligibility Criteria

The eligibility criteria included the following:

Population:

Included studies comprised adult participants with a clinical diagnosis of PD. Reviews with mixed neurological populations were excluded unless PD-specific data were reported independently.

Intervention:

The primary modality was defined as structured Resistance Training (RT) or PRT, requiring force exertion against an external load (machines, free weights, or resistance devices).

Comparator:

Controls were categorized as active (e.g., aerobic exercise, balance training, or physiotherapy) or passive (e.g., usual care, sham interventions, data bank, or wait-list data).

Outcomes:

The primary outcome included muscle strength. Secondary outcomes included muscle power, functional balance and mobility (e.g., Berg Balance Scale, Timed Up and Go test (TUG)), gait (e.g., walking speed, step length), and patient-reported outcomes, such as QoL.

To synthesize the efficacy of PRT, outcome data were extracted from the results of existing meta-analyses and systematic reviews. These data were then qualitatively categorized based on the reported statistical significance and directionality (as detailed in Table S9):

- Significant: A statistically significant improvement was reported in the intervention group compared to control.
- No Significant Difference: No statistical difference between groups was reported in the intervention group compared to control.
- Positive Trend (+Effect): Beneficial directionality was reported, but results did not reach statistical significance or were inconsistent across primary trials.
- Not Applicable (N/A): Outcome measure was not reported for PRT within the respective review.

### 2.3. Search Strategy and Selection Process

The literature search across databases and the application of predefined selection criteria were conducted independently by the primary author. In cases of ambiguity regarding study eligibility, a formal consultation was undertaken with two independent experts—one in adapted physical activity and one in rehabilitation sciences—until a consensus was reached. To ensure a comprehensive synthesis, the literature search was conducted across five major academic databases: PubMed, Cochrane, ProQuest, EBSCO, and Google Scholar. The search strategy employed a combination of MeSH terms (e.g., Resistance Training, Parkinson’s Disease) and free-text keywords (e.g., Strength Training, Functional Capacity, Systematic Reviews, Literature Reviews). To ensure relevance, the search was limited to systematic reviews and meta-analyses available as full text in English, covering the period from database inception through 1 May 2025. Additional details on the literature search across the databases are available in Table S3.

The systematic search across five academic databases initially identified 149 records. After the removal of 42 duplicates, 107 unique records remained for screening. Following the initial screening of titles and abstracts, 21 records were excluded. Upon full-text evaluation of the remaining 86 reports, an additional 48 reports were excluded for not meeting the inclusion criteria. 38 systematic reviews met the eligibility criteria and were included in this umbrella review. To ensure full transparency in accordance with PRISMA 2020 guidelines, a comprehensive list of all 69 excluded records and the specific rationales for their exclusion are provided in Table S2.

### 2.4. Selection of Primary Clinical Studies

To provide a highly specific and comprehensive analysis of the effects of PRT on PD, a systematic extraction of primary clinical studies from the included reviews was conducted.

Studies were included in the final analysis matrix if they met the following strict criteria:

- Population and Language: Primary studies involving patients diagnosed with PD, with the full-text article published in English.
- Frequency—To capture the most impactful and extensively evaluated evidence in the field, primary studies demonstrating a higher frequency of inclusion across the selected systematic reviews were chosen as the core sample, provided they met the strict methodological criteria below.
- Intervention Specificity: The intervention had to consist exclusively of isolated strength or PRT. Studies examining PRT combined with ergogenic aids or nutritional supplements (e.g., Creatine) were excluded to isolate the mechanical and physiological effects of the exercise itself.
- Comparison of Mechanical Stimuli: To understand the impact of specific mechanical stimuli and training adaptations, we purposely included studies that compared different modalities of strength training against each other (e.g., traditional PRT vs. eccentric training, or PRT with vs. without instability).
- Comparators (Control Groups): Studies must have included a valid clinical control group, either active (e.g., alternative non-resistance exercises) or passive (e.g., standard care, educational lectures). Studies utilizing wait-list controls, historical controls from tissue/data banks, or single-arm trials with only a before/after design were excluded due to high risk of bias.
- Outcomes: The studies had to evaluate at least one of the following primary or secondary outcomes: muscle strength, functional performance (e.g., motor signs, steps/gait velocity), balance, or QoL.
- Multiple Publications from a Single Protocol: In cases where multiple articles were published based on the same clinical trial or cohort, all relevant studies were deliberately included in the matrix. This approach was chosen to present a complete, holistic clinical picture of the protocol’s pleiotropic effects across various distinct outcomes (e.g., motor skills, sleep, and QoL).

The frequency table of the study’s citation count across the reviews, with the specific justifications for the exclusion of studies, is in Table S10.

**Figure 1:**
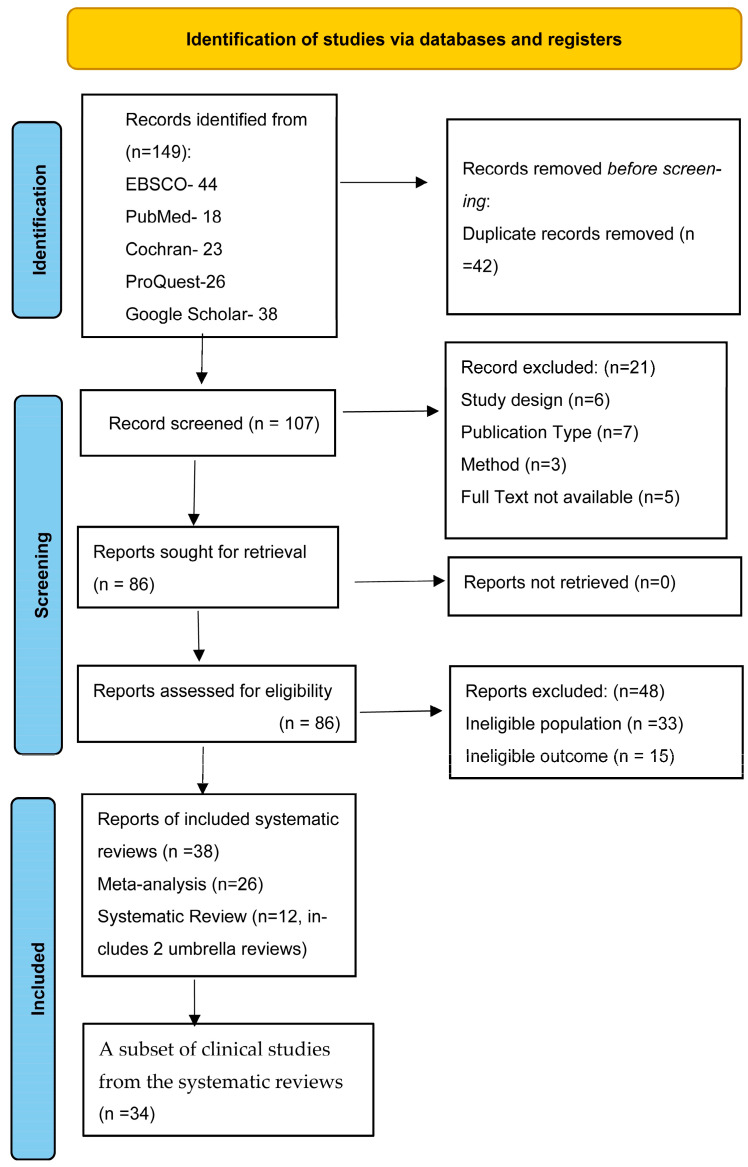
Flow diagram of report-selecting process.

The study selection process is detailed in the PRISMA flow diagram (Figure 1).

### 2.5. Evaluative Framework for Intervention Adherence

To investigate the variability in clinical efficacy, PRT protocols from 34 purposefully selected clinical studies were evaluated against a three-tiered methodological framework:

- FITT-VP ACSM: Assessing fundamental exercise prescription compliance (Frequency, Intensity, Time, Type, Volume, Progression).
- Integrated Guidelines: Evaluating the synthesis of FITT-VP with population-specific supplementary needs, such as motor complexity and functional tasks.
- Principles of progression: Measuring mechanistic fidelity through adherence to Progressive Overload, Variation, and Specificity.

### 2.6. Statistical Coding

The methodological quality assessment and the systematic coding of data were executed exclusively by the primary researcher, acting as a specialist in sports science. The researcher established the coding criteria based on validated scientific frameworks and consulted with two expert reviewers to resolve any cases of ambiguity, ensuring the precision and reliability of the analytical process.

A specialist in sports science, who is the first author, systematically evaluated and coded each intervention using a 3-point adherence scale: 2 (high), 1 (uncertain/partial), and 0 (non-adherent) [11].

Adherence patterns across the included literature were synthesized using descriptive statistics. Following the methodology established by Cui et al. [11], a threshold of ≥70% was utilized to categorize an intervention as demonstrating “high adherence,” while scores <70% were classified as “low or uncertain adherence.”

### 2.7. Evaluative Frameworks

The following three tables define the criteria used to code the interventions in the 34 clinical studies.

To further clarify the application of this coding system, a detailed example involving the study by Cherup et al. [1] is provided in Table S4. The criteria of the FITT-VP ACSM are detailed in Table 1. 

This framework assesses basic exercise prescription compliance to ensure a standardized physiological dose.

While some of the contemporary literature estimates exercise dose using Metabolic Equivalents (METs) [21,22,23], this review intentionally utilizes the specific ACSM FITT-VP principles for assessment. METs often misclassify intensity in neurological populations due to individual variances in body weight, biological sex, and mechanical efficiency (ACSM’s Guidelines [10,24]). Cui, Li, Yue et al. [11] investigated the effect of exercise dose on individuals with PD and found that studies with exercise interventions that had a high adherence to the FITT-VP ACSM showed a more significant improvement in motor function, mobility, and quality of life, but not in balance. Assessing adherence to the ACSM’s recommendations and using this type of coding system also appears in other studies designated for various populations [11,12,13,14,15,16,17,25].

Details on the adherence scores of the subset clinical studies to the FITT-VP are available in Table S5. The criteria of the Integrated Guidelines are detailed in Table 2.

Details on the adherence scores of the subset clinical studies to the Integrated guidelines are available in Table S6. The criteria of the Principles of Progression are detailed in Table 3.

Details on the adherence scores of the subset clinical studies to the principles of progression are available in Table S7.

### 2.8. Quality Assessment and Risk of Bias Assessment

The methodological quality of the included Systematic Reviews was evaluated using the Risk of Bias (RoB) tool—AMSTAR 2 [26]. The assessment was primarily conducted by the primary researcher with a secondary review of the scoring performed by two independent experts. This validated tool assesses 16 domains, with seven of them considered critical domains:

1. Protocol registered before commencement of the review (item 2);
2. Adequacy of the literature search (item 4);
3. Justification for excluding individual studies (item 7);
4. Risk of bias from individual studies being included in the review (item 9);
5. Appropriateness of meta-analytical methods (item 11);
6. Consideration of risk of bias when interpreting the results of the review (item 13);
7. Assessment of the presence and likely impact of publication bias (item 15).

## 3. Results and Synthesis

### 3.1. Results of Adherence of the Subset Clinical Studies to the Frameworks

The methodological evaluation focused on a subset of 34 clinical trials identified within the 38 systematic reviews and meta-analyses. Adherence was measured against three frameworks: general ACSM recommendations, the principles of progression, and the integrated guidelines specifically tailored for PD. The adherence scores for the frameworks are detailed in Table 4. 

Within the 26 studies utilizing an active control group, PRT demonstrated clinical superiority in 57% (*n* = 15) of the trials. When referring to the effect on specific functional or balance outcomes, PRT demonstrated clinical superiority in 46% (*n* = 12) of the trials. Among these superior outcomes (functional or balance), a notable level of adherence was observed across all evaluative frameworks: 58% (*n* = 7) adhered to the principles of progression, 66% (*n* = 8) followed the general FITT-VP ACSM recommendations, and 75% (*n* = 9) met the FITT-VP criteria integrated with integrated guidelines. Additionally, 2 of the 26 active comparison studies evaluated three distinct PRT methods and found both interventions to be similarly effective [1,27,28].

In contrast, 42% (*n* = 11) of the active comparison studies reported that PRT was either equivalent or inferior to the control group, but this rate increased to 54% (*n* = 14) when referring to the effect on specific functional or balance outcomes. Notably, 100% of these non-superior studies failed to meet the 70% threshold for adherence to the principles of progression. While 78% (*n* = 11) of these trials maintained high adherence to general ACSM recommendations, only 50% (*n* = 7) adhered to the integrated guidelines.

Regarding the eight studies that compared PRT to a passive control group, the intervention was found to be effective in 100% of the cases. In this group, adherence rates varied: 62.5% (*n* = 5) followed the principles of progression, 87.5% (*n* = 7) met the integrated guidelines, and 100% (*n* = 8) adhered to general ACSM recommendations.

### 3.2. Synthesis of the Adherence to FITT-VP, Integrated Guidelines, and Principles of Progression

The comparative analysis of 26 studies employing an active control group identifies a distinct “Methodological Threshold” for clinical superiority. While general exercise prescription (FITT-VP) remained consistently high across all trials (66% vs. 78%), a stark divergence emerged regarding progression; only the superior interventions crossed the 70% threshold, whereas non-superior trials failed it entirely. The comparative adherence and the outcomes matrix are detailed in Table 5. 

To account for the methodological heterogeneity observed across trials, a subgroup synthesis was conducted. Within the subset of studies where PRT was superior (46%), 58% of protocols maintained high adherence to the principles of progression. In contrast, in the 54% of studies where PRT was either equivalent or inferior to the active control, none of the protocols met the high adherence threshold for progression. This may indicate that mechanistic growth and specificity are important drivers of competitive superiority, and the heterogeneity is closely linked to the lack of physiological fidelity and progression in the intervention designs.

Superior outcomes were also strongly linked to the Integrated Guidelines, with 75% adherence in the superior group versus only 50% in the non-superior group. This suggests that failing to tailor PRT to the specific clinical needs of Parkinson’s patients (e.g., functionality, balance, progression) significantly dilutes the intervention’s efficacy.

The results further clarify why PRT appears universally effective in simpler study designs. In the eight studies utilizing a passive control, 100% found PRT to be effective, while 62.5% of them utilized high-level progression. This demonstrates that while a baseline PRT protocol is sufficient to beat inactivity, it is fundamentally insufficient to beat another active modality. To achieve clinical superiority compared to an active control group, it seems that PRT protocol should consist of functional exercises and progressive overloads.

### 3.3. Results of Outcomes in the Systematic Reviews and Meta-Analyses

A comprehensive analysis demonstrates that the impact of PRT on the outcomes ranges from “significant” to “positive” effects. Research designs typically compared PRT to passive or active control groups [6,7,59,60,61] or evaluated PRT as a standalone intervention versus a multi-modal protocol [59,62]. Three reviews utilized the Surface Under the Cumulative Ranking (SUCRA) to determine intervention hierarchy, noting that while PRT is effective, its ranking varies by outcome (e.g., highly effective for strength but sometimes secondary for gait) [61,63,64]. Three reviews assessed exercise dosage via MET-min/week, identifying optimal thresholds for symptom relief (e.g., 750–1000 MET-min/week for RT) [21,22,23]. Eighteen reviews explicitly observed that PRT improves muscle strength, torque, and volume (13 significant, 5 positive trends). Fifteen reviews found that PRT positively influences functional capacity and mobility (10 significant, 5 positive trends), specifically improving markers like the Timed Up and Go (TUG), Sit to Stand, and the 6-Minute Walk Test (6MWT); seventeen reviews confirmed that PRT improves motor signs (nine significant and eight positive trends) as measured by the Unified Parkinson Disease Rating Scale (UPDRS). Additionally, balance reported positive outcomes in 16 reviews each (11 significant and 5 positive trends for both). Sixteen reviews noted improvements in gait (11 significant and 5 positive trends), including gait velocity [65,66], walking velocity [22], and stride length; fifteen reviews reported improvements in QoL following PRT interventions (8 significant, 7 positive trends), and freezing of gait (FOG) was addressed in 4 reviews, all reporting significant findings. Regarding the study comparators, the majority of the synthesized literature (*n* = 32) included trials comparing PRT to both active and passive control groups. Four reviews focused solely on active comparators, one focused on passive controls, and one review specifically compared muscle mechanical function in people with PD to healthy controls [9]. Regarding comparative efficacy, the synthesis of the 38 included reviews reveals that PRT is reported as superior to other interventions in 14 reviews. In contrast, 23 reviews found no significant difference, reporting that PRT is comparable (equally effective) to other active physical interventions. Only one review, which focused on non-motor outcomes, reported PRT to be less effective than its comparator [67].

Detailed information on included reviews and outcome measures is available in Table S8 and S9, respectively.

### 3.4. Synthesis of PRT Interventions in the Systematic Reviews and Meta-Analyses

#### 3.4.1. Differential Outcomes of PRT Compared to Other Interventions

This synthesis of findings from systematic reviews and meta-analyses on PRT in PD highlights its often-inconclusive impact, especially when compared with active control groups. The collective literature examining PRT in PD presents a nuanced perspective on its efficacy. Although PRT consistently yields significant benefits compared to non-active controls, its superiority over other active interventions frequently remains inconclusive.

For example, reviews by Roeder et al. [62] and Saltychev et al. [68] reported that PRT improved muscle strength but failed to provide additional benefits compared to other active modalities. This observation is mirrored in subsequent studies, which determined that, while PRT enhanced outcomes like muscle strength, physical function, mobility, balance, and QoL, it was not demonstrably more effective than other active interventions such as yoga, Tai Chi, or gait and balance training [6,7,69]. Similarly, Uhrbrand et al. [70] indicated that the effect of PRT on QoL is inconsistent.

In contrast, other reviews have identified distinct outcomes associated with specific interventions. For example, Lima et al. [71] suggested PRT is effective for enhancing walking capacity, although these benefits did not extend uniformly across all physical performance measures. Similarly, Tambosco et al. [72] and Gamborg et al. [73] found that PRT positively influenced strength and functional capacity, whereas aerobic exercise often yielded superior gains in cardiorespiratory fitness. Song et al. [66] reported that both PRT and aerobic exercise positively affected motor and gait performance (UPDRS and gait velocity); however, aerobic exercise had a significant effect on motor and gait performance, while PRT demonstrated a significant effect on balance (Mini-BESTest). Furthermore, research by Tonkin et al. [67] and Padilha et al. [74] noted varied efficacy across motor and non-motor symptoms for different exercises, such as Tango and combined training. Tonkin [67] identified combined resistance and body-weight functional training as the least effective for non-motor experiences, potentially due to the short exercise duration (48 h) and the advanced disease stage of the participants. Karpodini et al. [75] indicated that rhythmic cueing and dance improved gait and motor symptoms, whereas PRT specifically improved the TUG test and QoL outcomes. Zhang et al. [61] also noted that effectiveness varied across different exercises and outcomes. Tonkin et al. [67] identified Tango and mixed treadmill training as most effective for non-motor experiences. Padilha et al. [74] highlighted that PRT, combined exercises, and specific activities improved both motor and non-motor outcomes, while aerobic and sensorimotor exercises primarily improved motor outcomes.

A key finding in the literature is that while some systematic reviews distinguish interventions by their specific outcomes, others suggest that different factors are more critical to the results. Some reviews emphasize that the dose and intensity of a training protocol are crucial determinants of its effect. For instance, Zhou et al. [64] suggested that short-duration, high-intensity PRT or aerobic interventions yielded better motor symptom improvements than other intensities and durations. Lima et al. [71] found that only a short duration of PRT improved strength.

Álvarez-Bueno et al. [63] also suggested more demanding interventions like PRT, endurance, and dance as the most effective. Similarly, recent dose–response analyses indicate varying effectiveness across different exercises (aerobic, PRT) at different doses, though these do not consistently reach the minimal clinically important difference [21,22].

Other systematic reviews and meta-analyses suggest that task-specific training is a vital factor for improving outcomes. For example, Tilman et al. [76] reported that PRT significantly enhanced strength but had no observable effects on balance and gait, suggesting it should be combined with balance and task-specific training for comprehensive benefits. Palucci et al. [77], in an umbrella review, suggested that while PRT positively impacts strength, it should be combined with balance training, postural control, and other exercises to preserve cardiorespiratory fitness and endurance in daily activities. Palheta De Lima et al. [78] recommended PRT as an effective alternative for improving motor performance and, when combined with balance training, for significantly improving balance and postural parameters.

#### 3.4.2. Significant Effect of PRT Compared to Other Interventions

A limited number of meta-analyses and reviews present contrasting evidence, suggesting PRT offers notable benefits in improving muscle strength and reducing functional impairments when compared to other physical interventions. Brienesse & Emerson [79] indicated that PRT has a beneficial effect on strength and functional performance, but noted the limited amount of robust literature on the topic. Lamotte et al. [80] found that PRT improved strength and motor signs, with potential benefits for functional outcomes, when compared to standard care, placebo, and other active groups. Chung et al. [81] recommended moderate-intensity PRT (2–3 times/week for 8–10 weeks) for gains in muscle strength, balance, and motor symptoms in PD, emphasizing progressive overload principles.

X. Li et al. [65] further showed that lower limb PRT improved leg strength, QoL, and gait performance (fast gait velocity, TUG, freezing of gait, but not stride length). Chamberlain-Carter & Jackson et al. [82] observed that PRT improved strength and had some effect on reducing falls and improving QoL. Zhaoli et al. [83] concluded that PRT significantly improved balance, mobility, and walking ability, though not stride length or walking speed. A systematic review and meta-analysis by Yang & Wang [60] revealed that PRT plays a positive role in rehabilitation for individuals with PD, particularly in improving muscular strength and QoL. The review also noted that PRT could, to some extent, reduce freezing of gait [60]. Controversially, Ramazzina et al. [84] noted that PRT improved physical parameters and QoL compared to active controls, but a correlation between strength and these improvements could not be established due to insufficient strength assessment.

### 3.5. Results of Outcomes in the Subset Clinical Studies

The analysis of a subset of 34 clinical studies reveals a diverse landscape of comparative designs and therapeutic outcomes. Among these, eight studies utilized a passive control group for comparison, while twenty-six studies evaluated PRT interventions against various active control groups. Notably, within the twenty-six active comparison trials, nine specifically compared two distinct methodologies of PRT to determine relative efficacy. The clinical impact of PRT was measured across several key physiological and functional domains: Eleven studies demonstrated that PRT is effective for increasing muscle strength, and another eight studies reported significant improvements in overall physical capacity. Eight studies established that PRT positively influences balance, mobility, and postural control. Therapeutic gains in gait were identified in four studies, while an additional three studies found PRT to be effective in reducing the rate of falls. Three studies reported improvements in motor signs, and five studies indicated enhanced QoL following the intervention. Finally, one study specifically demonstrated effectiveness in reducing the severity of freezing of gait.

### 3.6. Synthesis of the PRT Interventions in the Subset Clinical Studies

This summary presents detailed findings from the individual clinical studies that contributed to the evidence base for the previously discussed systematic reviews. All studies examined PRT interventions in individuals with PD (specific characteristics are detailed in Table S11). This synthesis examines findings from clinical studies on PRT in PD, starting with comparisons to non-active groups, moving to differential outcomes among active interventions, and concluding with instances where PRT demonstrated unique advantages.

#### 3.6.1. Consistent Benefits of PRT Compared to Non-Active Groups

Clinical studies corroborate the trend observed in meta-analyses: PRT yields significant effects when compared to non-active control groups. Allen et al. [52] found that minimally supervised PRT, combined with balance training, reduced fall risk and freezing of gait risk, while improving sit-to-stand performance and muscle strength. Hass et al. [53] further supported these motor improvements, reporting that PRT improved posterior center of pressure displacement and initial stride length and velocity. Their program notably utilized a multi-directional seated ankle TheraBand protocol alongside full-body exercises. Training focused on leg muscle power effectively improved leg power and strength, with potential beneficial effects also noted on mobility, balance, and falls [37]. Beyond core motor function, low-volume PRT was found to enhance overall physical capacity [36]. Strength training also significantly improved inspiratory and expiratory muscle strength and QoL [38], while other PRT programs reduced anxiety symptoms and boosted QoL [35]. Further confirming this, PRT protocol was found to reduce depressive symptoms, QoL, and functional performance [58].

Protocols with PRTI demonstrated particularly robust efficacy. Both PRT alone and PRT with instability significantly improved muscle strength compared to a passive control group; however, the PRTI group also enhanced mobility, motor signs, cognitive impairment, and QoL [31]. Integrating instability optimized neuromuscular adaptations, providing a clear physiological mechanism for the significant gains observed in mobility, motor signs, and QoL [32]. PRTI was specifically recommended for improving balance and fear of falling, with gains strongly associated with simultaneous improvements in cognitive function [33].

More recent studies confirm and expand this range of benefits: PRT may reduce bradykinesia and improve diverse functional performance outcomes (e.g., TUG, 30 s chair stand, 10 m walk test) [4]. 

#### 3.6.2. Differential Outcomes of PRT Compared to Other Interventions

Foundational mobility and balance programs frequently match or outperform PRT in core functional measures. For example, studies found no significant differences between PRT and balance training in improving postural control and freezing of gait [50,51]. F. Li et al. [49] found Tai Chi improved functional capacity and reduced falls when compared to stretching but was not specifically superior to PRT. Both PRT and movement strategy training significantly reduced fall rates when combined with falls prevention education [42].

In long-term studies, both PRT and a modified fitness count program comparably improved functional performance over 24 months [41]. Programs like power training and high-speed yoga yielded similar, significant improvements across motor symptoms, strength, power, and balance [43]. However, a direct comparison between PRT and power training found both improved strength and power without significant differential effects, yet neither improved overall functional performance [27]. Conversely, S. M. Santos et al. [85] observed improvements in postural control exclusively within the balance training group when compared to PRT.

The literature suggests that the benefits of PRT in isolation often fail to translate fully to daily function and gait parameters. Shulman et al. [54] observed that both PRT and treadmill training improved Gait. While PRT improved strength and treadmill training improved fitness, these specific gains did not translate into improvements in daily function [54].

When PRT is modified to increase motor complexity, its efficacy appears to improve. The addition of PRTI enabled improvements not only in muscle strength (like PRT alone) but also uniquely enhanced mobility, motor signs, cognitive impairment, and QoL [31]. This highlights that challenging the neuromuscular system beyond pure load is paramount. Shen & Mak [55] observed that, while both balance training with augmented feedback and PRT improved stability limits, single-leg stance time, and gait speed, only the balance group sustained improvements in balance confidence and stride length at 12 months. Furthermore, over 15 months, the specialized balance group ultimately demonstrated fewer fallers and a reduced fall rate compared to the PRT group [56]. The literature also indicates complementary effects of exercise and medication on bodily functions, suggesting benefits are derived not strictly from the exercise modality alone, but from the integration of care [1].

#### 3.6.3. Significant Effect of PRT Compared to Other Interventions

A subset of clinical studies specifically highlights the distinct effectiveness of PRT, suggesting that training parameters like intensity, duration, and task-relevance are crucial determinants of desired outcomes. PRT combined with balance training was found to improve both muscle strength and balance [30]. Over 24 months, PRT demonstrated unique efficacy; it was the only intervention—when compared to a modified fitness count program—to improve motor signs [40]. Maximal strength PRT may effectively improve skeletal muscle force-generating capacity, efferent neural drive, and functional performance, leading researchers to advocate for high-intensity strength training as an adjunct therapy [39].

The most significant benefits appear when PRT is deliberately adapted for complexity. Silva-Batista et al. [29] showed that PRT integrated with instability led to significant clinical improvements and enhanced brain plasticity in individuals with freezing of gait. In a comparison with traditional motor rehabilitation, both PRTI and the traditional approach increased gait speed and stride length; however, only the PRTI group showed improved gait automaticity and attentional set-shifting, demonstrating that its progression prioritized motor complexity [34].

Different PRT modes can target specific clinical features. Hypertrophy and functional training modes of PRT were found to be equally effective in improving functional capacity, balance, and muscle strength and were suggested to target freezing of gait and motor symptoms, respectively [28]. Finally, Marie Domonceau & Didier Maxuet [45] noted that PRT improved most peak torque measures, peak workload, and 6MWT, although other fitness measures did not consistently translate into better mobility and QoL.

These comparative studies collectively suggest that PRT’s effectiveness is highly dependent on factors such as intensity, duration, and task-specificity. This is evidenced by the structural variations in successful programs: one study notably incorporated PRT with balance training [30], two trials integrated PRT with instability training [29,34], and a third study directly compared two PRT protocols emphasizing either functional or multi-joint exercises [28]. This heterogeneity in design highlights an evolving academic understanding: maximizing desired functional outcomes requires deliberately manipulating specific training parameters and task relevance.

### 3.7. Results of the AMSTAR 2 Quality Assessment of RoB Assessment

The detailed AMSTAR2 quality appraisal of the included systematic reviews is provided in Table S12. A summary of this detailed quality appraisal is presented in Table 6.

The methodological quality of the included systematic reviews was evaluated using the AMSTAR 2 tool. The methodological quality assessment reveals a stark contrast between high adherence to foundational review structures and significant deficiencies in transparency measures. All 38 reviews (100%) successfully defined their research questions using PICO components, and 97% adequately justified their study design selection. However, critical rigor was inconsistent; nearly half of the reviews (47%) failed to register a protocol a priori, raising concerns regarding potential selective reporting bias. Furthermore, transparency was largely lacking in key domains: comprehensive search strategies were achieved by only 13% of the reviews, and the reporting of excluded studies was negligible, with only three reviews—Ernst et al. [7], De Almeida et al. [69], and Song et al. [66]—providing a complete list with reasons for exclusion. Most critically, funding transparency was virtually absent, as Ernst et al. [7] was the sole study (3%) to report the funding sources of the primary studies included in their analysis.

## 4. Discussion

### 4.1. The Link Between Methodology and Efficacy

This umbrella review synthesizes a comprehensive body of evidence regarding PRT for individuals with PD. While the literature consistently supports the superiority of PRT over non-active control groups, data concerning its efficacy compared to other active interventions remains equivocal. Furthermore, while the effect of PRT on muscle strength is well established compared to active controls, its impact on functional performance also presents a mixed pattern. This review suggests that the lack of consensus regarding PRT’s superiority over alternative active interventions, such as balance, aerobic, or mind–body training, could be potentially linked to inconsistent adherence to the established principles of progression within the research literature. Although progression, intensity, and specificity are well delineated within existing frameworks (FITT-VP ACSM, Integrated Guidelines, and principles of progressions), their partial implementation in PRT protocols has likely contributed to these heterogeneous outcomes. Additionally, distinct from other frameworks, the principles of progression emphasize the importance of tempo, a full range of motion, and exercise variation.

### 4.2. The Impact of Insufficient Adherence

A critical examination of the included systematic reviews and clinical studies reveals that PRT protocols frequently fail to adhere to the three essential principles of performance progression: progressive overload, variation, and specificity, which are embedded within the FITT-VP ACSM and Integrated Guidelines frameworks.

#### 4.2.1. Detailed Evidence from Systematic Reviews and Meta-Analysis

This methodological deficiency is evident in the systematic review literature, where researchers like Ernst et al. [7] and Karpodini et al. [75] explicitly noted that certain studies failed to report the intensities and volumes of their interventions. This lack of transparency obscures the dose–response relationship. Furthermore, the literature stresses that adequate task-specificity is essential for translating strength gains into functional improvements. Tillman et al. [76] recommended that PRT be performed in conjunction with balance and task-specific functional training, while Coste et al. [86] indicate that any exercise modality may improve motor symptoms, as long as it is periodized and systematized. Gollan et al. [6] suggested that the lack of beneficial effects of PRT over alternative interventions could stem from protocol overlap, or from low-intensity PRT protocols inadvertently inducing metabolic stimuli—similar to balance training—rather than the requisite mechanical stimuli. In the study of De Almeida et al. [69], the lack of beneficial effects of power training compared to PRT may be attributed to lower training loads in the power training interventions, which may also explain the lack of improvements in gait speed tests.

#### 4.2.2. Detailed Evidence from Clinical Studies

Clinical evidence confirms the premise that a failure to implement stringent overload and specificity correlates directly with minimal functional improvement:

- Insufficient Overload: In a 24-week study comparing PRT to Tai Chi, the PRT intervention’s minimal load progression (only 1–4 kg) likely explains the minor strength and functional gains relative to the Tai Chi group [49]. This disparity in load seemingly compromised the benefits of the PRT, even though in the inclusion of gait-specific exercises like side/forward steps and lunges, which did yield notable improvements in stride length. Consequently, this study demonstrates merely 50% adherence to the principles of progression [49]. Similarly, Morris et al. [42] utilized a progression based on a marginal increase in weight (2% of body weight), increasing sets to three, or increasing repetitions to 15 over only eight weeks, suggesting a limited mechanical stimuli. The marginal load increments in this PRT protocol were applied to functional exercises that closely mirrored those performed by the active control group [42], resulting in a relatively low adherence to the principles of progression (66%). Furthermore, while Shen & Mak [55,56] incorporated progressive loading in seated exercises, the progression of their functional exercises relied on increasing repetitions, which theoretically induced metabolic rather than mechanical stimuli.
- Inappropriate Progression Variables: Even when progression was prescribed, its implementation was often conservative; Dibble et al. [10] found no significant differences between concentric and eccentric lower-extremity PRT protocols despite maintaining progression (RPE of 13). In Dibble’s study [1], the progression of training duration extended up to 30 min, prompting the consideration of whether the intervention functioned more as an aerobic stimulus than a genuine PRT regimen. Moreover, even when muscular strength improved, a failure to achieve distinct neuromuscular stimuli resulted in non-superiority. Cherup et al. [45] found no significant differential effects between PRT and power training; both groups performed the same exercises without functional tasks. While one used volitional fatigue and the other focused on power output, the achievement of peak rate of force development requires a foundation of maximal strength coupled with the intent to move a load with maximal velocity [87]. Because both protocols mandated 10 repetitions, they likely imparted comparable physiological stimuli, consequently leading to equivalent outcomes and allowing adherence score (66%) to principles of progression. Conversely, Strand et al. [28], who achieved high adherence scores across all frameworks, compared two strength and power protocols, one integrated with hypertrophy training and the other with functional training. While both modalities enhanced strength and functional capacity, only the functionally oriented group demonstrated improvements in FoG and motor symptoms. A plausible explanation for the divergent results between the two studies [27,28], beyond variations in outcome measures, may be the inclusion of functional exercises and the precise magnitude of the applied stimuli. Strand et al. [28] differentiated the intensity, tempo, and volume of each method, thereby emphasizing the targeted physiological stimuli.
- Lack of specification: Domonceau & Maxuaet [45] reported that while PRT enhanced walking capacity, neither PRT nor aerobic improved mobility and QoL. The authors explicitly mentioned that the interventions were not specified to improve these outcomes [45]. Additionally, Schilling et al. [48] found that a PRT protocol, consisting exclusively of machine-based exercises, improved strength but failed to improve functional mobility. The authors noted that these findings contrast with those of Dibble et al. [46,47], who observed performance improvements following a PRT protocol. Beyond potential differences in clinical characteristics of the PD participants, it should be noted that the protocols utilized by Dibble et al. [46,47] incorporated balance exercises and treadmill walking alongside eccentric ergometry, thereby possibly satisfying the requirement of “specificity”. This methodological distinction may also contextualize the low adherence scores (66%) assigned to these three studies, despite evidence from Dibble’s studies that PRT can affect functional performance [46,47].

### 4.3. The Impact of High Adherence

Conversely, evaluation of studies that report significant functional improvements and strength gains reveals a discernible pattern: effective PRT protocols tend to adhere to the core principles of progression.

#### 4.3.1. Detailed Evidence from Systematic Reviews and Meta-Analysis

Reviews that observed additional benefits of PRT, such as Brienesse & Emerson [79] and Chung et al. [81], noted that effective protocols consistently prescribed moderate to high intensities (ranging from 60–80% of 1 RM). Lamotte et al. [80] highlighted that all studies incorporated progressive loading, although specifics varied. Importantly, in the review of Yang & Wang [60], the studies that evaluate the effect of PRT on FoG successfully employed progressive loads and paid careful attention to exercise intensity [29,52,85].

#### 4.3.2. Detailed Evidence from Clinical Studies

Clinical studies confirm that when PRT protocols introduce adequate stimulus and specificity, they yield notable advantages:

- Longitudinal Progression: Although the study of Corcos et al. [40] failed to achieve high adherence to the principles of progression (66%), the PRT intervention was found superior to the mFC. A possible explanation could be that, although the grade of “specificity” was low (0—no adherence), since none of the PRT exercises challenged balance, it is the only study that observed PRT intervention during 24 months. This 24-month trial demonstrated that only the PRT group, using single and multi-joint exercises with progressive loads, achieved significant improvements in UPDRS III compared to a control group performing non-progressive balance-strengthening training [40]. A secondary analysis of this study showed that both methods were effective in improving functional performances [41].
- Overload Adaptation: Helgerud et al. [39] investigated a 5-week maximal strength training program consisting four sets of four repetitions at 90% of 1RM for the leg press and bench press exercises. Only the Maximal Strength Training group significantly improved skeletal muscle force-generating capacity, efferent neural drive, and functional performance (including stair climbing and the TUG test), while the control group performing submaximal training showed no such gains. Given that both protocols incorporated functional exercises and balance training, this study emphasizes the importance of progressive mechanical overload. Furthermore, the exceptionally high intensity utilized in this protocol accounts for its low adherence score to the FITT-VP ACSM (37.5%), while concurrently explaining the high adherence to the principles of progression (83%).
- Motor Complexity (Specificity): The integration of PRTI has proven particularly robust, with all the PRTI studies achieving high adherence scores across the three frameworks (83–100%). Silva-Batista et al. [31] found that while both PRT and PRTI improved strength, only the PRTI demonstrated comprehensive improvements in mobility, motor signs, cognitive impairment, and QoL. Vieira-Yano et al. [34] confirmed that prioritizing motor complexity (e.g., lunges with instability) was essential for improving gait automaticity and attentional set-shifting. Similarly, Hirsch et al. [30] found that PRT combined with balance is more effective than only balance in improving muscle strength and balance. The present review highlights that none of the PRT protocols consisted of functional exercises simultaneously with progressive loads, even in instances of personalized supervised training. Instead, functional exercises were executed with only marginal load increments [42,50,51,55,56], utilizing body weight [28,39], or implemented as an adjunct to balance [1,30,39,46,47], and treadmill [1,46,47].

### 4.4. Limitations

While the primary objective of this umbrella review is exclusively to analyze the methodological deficiencies in the implementations of PRT protocols in the PD literature and to evaluate how these deficiencies contribute to the inconclusive rehabilitation trends, it is imperative to acknowledge several limitations. Addressing these constraints within the existing literature and the current review methodology is essential to ensure a balanced, cautious academic perspective. First, as an umbrella review, our findings are inherently dependent on the reporting quality and transparency of the existing systematic reviews. Any omissions or inaccuracies in the secondary evidence base may have influenced the overall synthesis. Furthermore, the possible duplication of primary studies across the included reviews is a recognized limitation of this study design. A primary limitation of this umbrella review is that screening, data extraction, and quality assessment processes using the established frameworks were performed by a single reviewer. Although a comprehensive list of excluded studies and detailed scoring for each framework was maintained, the absence of an independent second reviewer inevitably increases the risk of subjective bias in the evaluation of intervention adherence and final conclusions. Furthermore, the 34 included clinical studies demonstrate significant methodological heterogeneity, which inherently complicates the synthesis of results. This review is constrained by considerable variability within the characteristics of the PD participants, as well as the diversity of the outcome measures employed. The analyzed interventions spanned a wide array of resistance training types, including cycle ergometer protocols, machine-based training, and exercises utilizing various resistance devices. Protocols varied between single-joint and multi-joint exercises, with some interventions combining PRT with balance training or comparing distinct PRT methods such as power, functional, hypertrophy, and traditional strength training. Extensive variability was observed in training duration, intensity, volume, and frequency across the study pool. A potential explanation for why this umbrella review did not identify higher progression adherence rates among studies demonstrating PRT superiority is that the nature of the control groups significantly influences the perceived efficacy of PRT. In studies comparing two different PRT methods (e.g., hypertrophy vs. functional), a “ceiling effect” often occurs. Since both interventions utilize highly effective resistance modalities, achieving statistically significant group differences becomes unlikely even when both arms yield substantial clinical improvements. In trials where the active control was a specialized, high-intensity intervention—such as treadmill training or augmented feedback balance training—the threshold for PRT to achieve “superiority” was markedly higher than in trials using general motor rehabilitation as a baseline. Finally, the evaluation of adherence to the proposed frameworks was occasionally hindered by incomplete reporting in the primary literature, such as RoM, Tempo, or fatigue levels. Combined with the inherent impossibility of double-blinding in exercise science, these factors may introduce performance or a placebo bias, particularly in subjective assessments such as QoL scales or the UPDRS.

## 5. Conclusions

The findings of this umbrella review suggest that the frequently perceived “inconclusiveness” regarding the efficacy of PRT in the PD literature may be, at least in part, predominantly a methodological artifact rather than an intrinsic failure of the exercise modality. These results present a plausible hypothesis that the effectiveness of PRT is not monolithic, but potentially dependent on the rigorous application of progressive overload, exercise variation, and task specificity.

The analysis of 34 clinical studies identifies a discernible association between adherence to PRT frameworks, most notably the principles of progression, and the observed effectiveness of PRT protocol in ameliorating PD symptomatology and enhancing the functional performances of people with PD. While these findings require future prospective validation, they suggest that future clinical protocols should strive to maintain a minimum of 70% adherence across three integrated frameworks (FITT-VP, Integrated Guidelines, and Progression Principles) to optimize therapeutic outcomes. Notably, the fact that 100% of the non-superior trials analyzed failed to reach the progression threshold further supports this hypothesis. This review underscores that true specificity should be viewed as an inherent component of the PRT framework. For PRT to translate into functional improvements, clinicians and researchers must execute systematic load increases specifically within task-relevant contexts.

Future research should shift from simple modality comparisons toward optimizing PRT protocols by integrating motor complexity alongside functional exercises with progressive mechanical loads. Such longitudinal and rigorously controlled trials are warranted to confirm whether these optimized protocols consistently lead to superior clinical outcomes in the PD population.

## Figures and Tables

**Table 1:** FITT-VP ACSM.

FITT-VP Principal	Recommendation for Persons with PD (ACSM Guidelines [10])
Frequency	2–3 days a week, with a rest period of at least 48 h between training sessions.
Intensity	30–60% of 1RM for beginners, and 60–80% for advanced exercisers. This intensity level should be based on the individual’s strength.
Time (Volume)	1–3 sets of 8–12 repetitions.
Type	Avoid free weights and use weight machines or resistance devices like bands or body weight.

FITT-vp-Frequency, Intensity, Time, Type, Volume, and Progression. ACSM-American College of Sports Medicine; PD—Parkinson’s Disease; RM—Repetition Maximum.

**Table 2:** Integrated Guidelines [10]. These guidelines assess “Clinical Specificity” or how well the program is tailored to the unique neuromotor needs of PD patients.

Resistance exercise recommendations for healthy older adults may also apply to persons with PD.
Progression should be individualized and customized to personal tolerance.
Programs should incorporate static, dynamic, and balance exercises within functional activities.
Activities should include stepping in all directions, walking with appropriate stride length, and sit-to-stand transitions.
Neuromotor training should advance motor complexity alongside quantitative parameters.
Increasing motor complexity can sometimes impede the progression of quantitative parameters.

**Table 3:** Principles of progression [8]. This framework assesses the physiological quality of the stimulus, which is essential for driving long-term neuromuscular adaptation and performance enhancement.

Principle	Requirement for High Adherence
Progressive Overload	Systematic and gradual increase in physiological stress (intensity, volume, or tempo)
Variation	Long-term alteration of training variables to prevent plateaus and optimize recovery
Specificity	Ensuring adaptations correspond to specific tasks (muscle groups, movement speed, and contraction type)

**Table 4:** PRT vs. control and adherence to frameworks (N = 34).

Study Group	N	FITT-VP (≥70%): N (%)	Integrated Guidelines (≥70%): N (%)	Progression Principles (≥70%): N (%)	Outcome Trend
Active Comparison Superior	12	8 (66)	9 (75)	7 (58)	PRT outperformed active control.
Active Comparison Non-Superior	14	11 (78)	7 (50)	0 (0)	PRT ≤ active control.
Passive Comparison Effective	8	8 (100)	7 (87.5)	5 (62.5)	PRT > no-treatment.

PRT—Progressive Resistance Training; N—Number of studies referring to the effect on specific functional or balance outcomes.

**Table 5:** The comparative adherence and outcomes matrix presents the evaluation of the PRT interventions in the included clinical studies against all three frameworks.

Study (Authors, Year)	FITT: %	Integrated Guidelines %	Progression %	Reported PRT Effect	Group Difference	Control Type
Silva-Batista et al. [29]	100	100	100	Improved clinical signs and brain plasticity	Superior	Active (TMR)
Hirsch et al. [30]	75	100	100	Improved muscle strength and balance	Superior	Active (Balance)
Silva-Batista et al. [31] ***	100	93.80	91.70	Improved mobility and motor signs	Superior *	Passive
Silva-Batista et al. [32] ***	100	93.80	91.70	Optimized neuromuscular adaptations	Superior *	Passive
Silva-Batista et al. [33] ***	100	93.80	91.70	Improved balance and reduced FoG	Superior *	Passive
Viera-Yano et al. [34] ***	87.50	87.50	91.70	Affected gait speed and automaticity	Superior *	Active (TMR)
Ferreira et al. [35]	87.50	87.50	83.30	Reduced anxiety and improved QoL	Effective	Passive
Leal et al. [36]	75	87.50	83.30	Improved physical capacity	Effective	Passive
Paul et al. [37]	87.50	87.50	83.30	Improved leg muscle power and strength	Effective	Active (Sham)
Alves et al. [38]	87.50	75	83.30	Improved inspiratory-expiratory strength and QoL	Effective	Passive
Helgerud et al. [39]	37.50	75	83.30	Improved force-generating capacity, efferent neural drive, and functional performance	Effective	Active (Low PRT)
Strand et al. [28]	100	87.50	83.30	Improved functional capacity and strength. F improved FoG and motor symptoms	Effective	Active (H vs. F)
Corcos et al. [40]	87.50	75	66.60	Affected UPDRS-III scores	Effective	Active (MFC)
Prodoehl et al. [41]	87.50	75	66.60	Improved functional performance	No Diff	Active (MFC)
Cherup et al. [27]	100	75	66.60	Reduced neuromuscular deficits	No Diff	Active (S vs. P)
Morris et al. [42]	87.50	75	66.60	Reduced rate of falls	No Diff	Active/Passive
Ni et al. [43]	100	75	66.60	Improved physical performance	No Diff	Active (Yoga)
Alessandro Carvalho et al. [44]	100	75	66.60	Affected disease symptoms and function	No Diff	Active (Aerobic)
Marie Domonceau & Didier Maxuet [45]	100	75	66.60	Affected training specificities compliance	No Diff	Active (Aerobic)
Viera De Moraes Filho et al. [4]	100	75	66.60	Reduced bradykinesia and improved function	Effective	Passive
Dibble et al. [46]	62.50	62.50	66.60	Improved muscle force, hypertrophy and mobility	Effective	Active (General Fitness)
Dibble et al. [47]	62.50	62.50	66.60	Reduced bradykinesia and improved muscle force production and QoL	Effective	Active (General Fitness)
Dibble et al. [1]	62.50	62.50	66.60	Affected body structure and function	No Diff	Active (Ecc/Con)
Schilling et al. [48]	75%	62.5%	66.6%	Affected only muscle strength	No Diff	Active (Standard Care)
Li et al. [49]	87.50	75	50	Reduced balance impairments and lowered the incidence of falls	Inferior to Tai Chi **	Active (Tai Chi and stretching)
Schlenstedt et al. [50]	75	50	50	Both groups improved posture	No Diff	Active (Balance)
Schlenstedt et al. [51]	75	50	50	Neither group reduced FoG	No Diff	Active (Balance)
Allen et al. [52]	75	75	50	Reduced risk of falls and improved FoG, sit-to-stand, and strength	Effective	Passive
Hass et al. [53]	75	62.50	50	Improved gait initiation	Effective	Passive
Shulman et al. [54]	75	50	16.60	Affected only muscle strength	Inferior **	Active (Treadmill)
Shen & Mak et al. [55]	50	50	16.60	Affected SLS time and gait speed	No Diff (Inferior only in 12-month carryover)	Active (Feedback)
Shen & Mak et al. [56]	50	50	16.60	Reduced falls	Inferior **	Active (Feedback)
Santos et al. [57]	62.50	50	0	Affected postural control	Inferior **	Active (Balance)
De Lima et al. [58]	100	100	83		Superior *	Passive

* Superior is limited only to studies that beat a different active modality and had high progression (≥70%). ** Inferior refers to PRT having less impact on specific functional/balance outcomes compared to the active control. *** Scores for studies with multiple experimental groups (e.g., PRT vs. PRTI) have been averaged to represent the study’s overall methodological adherence. FITT-VP—Frequency, Intensity, Time, Type, Volume; Integrated Guidelines—Integrate Supplementary Clinical Guidelines; Progression—Progressive Resistance Training; FoG—Freezing of Gate; QoL—Quality of Life; UPDRS—Disease Rating Scale Motor Subscale; RoM—Range of Motion; Ecc—Eccentric; Con—Concentric; SLS—Straight Leg Stand; S—Strength; P—Power; TMR—Traditional Motor Rehabilitation; MFC—Modified Fitness Count. A proportion of ≥70% designated high adherence, and <70% designated low or uncertain adherence. The detailed coding and adherence of the included clinical studies to the FITT ACSM is provided in Table S5, with additional coding and adherence to the Integrated Guidelines in Table S6 and to the principles of progression in Table S7.

**Table 6:** AMSTAR 2 quality assessment.

AMSTAR 2 Domain	Compliance (Yes)	Non-Compliance (No, Partial, N/A)	Key Insight
1. PICO Components	38 (100%)	0 (0%)	All reviews established clear research questions.
2. Protocol Registration	20 (53%)	18 (47%)	Nearly half of the reviews did not register a protocol a priori.
3. Study Design Selection	37 (97%)	1 (3%)	Almost all reviews justified their study design, with one exception.
4. Search Strategy	5 (13%)	33 (87%)	Most reviews only partially met the comprehensive search criteria (e.g., searching trial registries or grey literature).
5. Duplicate Selection	35 (92%)	3 (8%)	High adherence to a dual independent study selection was observed.
6. Duplicate Extraction	27 (71%)	11 (29%)	Approximately 30% of reviews did not perform data extraction in duplicate.
7. Excluded Study List	3 (8%)	35 (92%)	A major transparency gap was observed; only 3 reviews provided a full list of excluded studies with reasons.
8. Study Details	36 (95%)	2 (5%)	Most reviews provided adequate detail on the included studies.
9. RoB Technique	35 (92%)	3 (8%)	The majority used a satisfactory technique for assessing the risk of bias.
10. Study Funding	1 (3%)	37 (97%)	A critical weakness was observed: only 1 review reported the funding sources for the primary studies included.
11. Meta-analysis Stats	30 (79%) *	N/A	High compliance among studies where meta-analysis was applicable/performed.
12. RoB Impact on Stats	16 (42%) *	N/A	Less than half assessed the potential impact of risk of bias on the statistical results.
13. RoB in Interpretation	36 (95%)	2 (5%)	Most reviews accounted for the risk of bias when discussing their results.
14. Heterogeneity	37 (97%)	1 (3%)	Nearly all reviews investigated or discussed heterogeneity satisfactorily.
15. Publication Bias	24 (63%) *	N/A	Publication bias investigation was mixed or not applicable in many cases.
16. Conflict of Interest	37 (97%)	1 (3%)	High transparency regarding the review authors’ own conflicts of interest was observed.

* Note: Percentages for items 11, 12, and 15 are calculated based on the total number of reviews (*n* = 38), but these items include “N/A” responses where meta-analysis was not performed.

## Data Availability

No new data were created or analyzed in this study. Data sharing is not applicable to this article.

## Supplementary Materials

The following supporting information can be downloaded at https://www.mdpi.com/article/10.3390/jfmk11020178/s1.

## Abbreviations

The following abbreviations are used in this manuscript:

PD	Parkinson’s Disease
FITTV-VP	Frequency, Intensity, Time, Type, Volume, and Progression
ACSM	American College of Sports Medicine
QoL	Quality of Life
PRT	Progressive Resistance Training
PICO	Participants, Intervention(s), Comparator(s), and Outcome(s)
PRISMA	Preferred Reporting Items for Systematic Reviews and Meta-Analysis
RT	Resistance Training
TUG	Time Up and Go
MET’s	Metabolic Equivalent
RM	Repetition Maximum
RoB	Risk of Bias
SUCRA	Surface Under the Cumulative Ranking
6MWT	6-Minute Walk Test
UPDRS	Unifies Parkinson Disease Rating Scale
FoG	Freezing of Gate
PRTI	PRT with Instability
