# Supplementary material for: Progressive Resistance Training in Parkinson’s Disease: An Umbrella Review Examining the Role of Methodological Adherence and Training Progression Principles in Clinical Outcome
Source: J Funct Morphol Kinesiol. 2026 Apr 28;11(2):178. doi: 10.3390/jfmk11020178 (PMC13214684; doi:10.3390/jfmk11020178)
Supplement: Supplementary file 1 [file jfmk-11-00178-s001.zip › jfmk-4184014-supplementary.pdf]

Table S1

Section and Topic	Item #	Checklist item	Location where item is reported
**TITLE**			
Title	1	Identify the report as a systematic review.	page 1
**ABSTRACT**			
Abstract	2	See the PRISMA 2020 for Abstracts checklist.	Page 1
**INTRODUCTION**			
Rationale	3	Describe the rationale for the review in the context of existing knowledge.	Page 1
Objectives	4	Provide an explicit statement of the objective(s) or question(s) the review addresses.	Page 1
**METHODS**			
Eligibility criteria	5	Specify the inclusion and exclusion criteria for the review and how studies were grouped for the syntheses.	Page 3
Information sources	6	Specify all databases, registers, websites, organisations, reference lists and other sources searched or consulted to identify studies. Specify the date when each source was last searched or consulted.	Page 4
Search strategy	7	Present the full search strategies for all databases, registers and websites, including any filters and limits used.	Page 4
Selection process	8	Specify the methods used to decide whether a study met the inclusion criteria of the review, including how many reviewers screened each record and each report retrieved, whether they worked independently, and if applicable, details of automation tools used in the process.	Page 4
Data collection process	9	Specify the methods used to collect data from reports, including how many reviewers collected data from each report, whether they worked independently, any processes for obtaining or confirming data from study investigators, and if applicable, details of automation tools used in the process.	Page 4
Data items	10a	List and define all outcomes for which data were sought. Specify whether all results that were compatible with each outcome domain in each study were sought (e.g. for all measures, time points, analyses), and if not, the methods used to decide which results to collect.	Page 3
	10b	List and define all other variables for which data were sought (e.g. participant and intervention characteristics, funding sources). Describe any assumptions made about any missing or unclear information.	Page 3
Study risk of bias assessment	11	Specify the methods used to assess risk of bias in the included studies, including details of the tool(s) used, how many reviewers assessed each study and whether they worked independently, and if applicable, details of automation tools used in the process.	Page 7

Section and Topic	Item #	Checklist item	Location where item is reported
Effect measures	12	Specify for each outcome the effect measure(s) (e.g. risk ratio, mean difference) used in the synthesis or presentation of results.	Page 5-6
Synthesis methods	13a	Describe the processes used to decide which studies were eligible for each synthesis (e.g. tabulating the study intervention characteristics and comparing against the planned groups for each synthesis (item #5)).	Appendix A.3- A.4
	13b	Describe any methods required to prepare the data for presentation or synthesis, such as handling of missing summary statistics, or data conversions.	Page 5, page 15
	13c	Describe any methods used to tabulate or visually display results of individual studies and syntheses.	Appendix B
	13d	Describe any methods used to synthesize results and provide a rationale for the choice(s). If meta-analysis was performed, describe the model(s), method(s) to identify the presence and extent of statistical heterogeneity, and software package(s) used.	Appendix C.1-C.3, page 13-15
	13e	Describe any methods used to explore possible causes of heterogeneity among study results (e.g. subgroup analysis, meta-regression).	Page 11-15
	13f	Describe any sensitivity analyses conducted to assess robustness of the synthesized results.	Page 5-6, page 13
Reporting bias assessment	14	Describe any methods used to assess risk of bias due to missing results in a synthesis (arising from reporting biases).	Page 15-16
Certainty assessment	15	Describe any methods used to assess certainty (or confidence) in the body of evidence for an outcome.	Page 11-16
**RESULTS**			
Study selection	16a	Describe the results of the search and selection process, from the number of records identified in the search to the number of studies included in the review, ideally using a flow diagram.	Page 5-6, figure 1
	16b	Cite studies that might appear to meet the inclusion criteria, but which were excluded, and explain why they were excluded.	Page 7, Page 9, Appendix A.2
Study characteristics	17	Cite each included study and present its characteristics.	Appendix A.3-4, Appendix B
Risk of bias in studies	18	Present assessments of risk of bias for each included study.	Page 7, page 15-16, Appendix D
Results of	19	For all outcomes, present, for each study: (a) summary statistics for each group (where appropriate) and (b) an effect estimate and its precision	Page 11-15,

Section and Topic	Item #	Checklist item	Location where item is reported
individual studies		(e.g. confidence/credible interval), ideally using structured tables or plots.	Appendix C 1-3
Results of syntheses	20a	For each synthesis, briefly summarise the characteristics and risk of bias among contributing studies.	Page 8-13
	20b	Present results of all statistical syntheses conducted. If meta-analysis was done, present for each the summary estimate and its precision (e.g. confidence/credible interval) and measures of statistical heterogeneity. If comparing groups, describe the direction of the effect.	Page 11-15, Appendix C 1-3
	20c	Present results of all investigations of possible causes of heterogeneity among study results.	Page 11-15, Appendix C 1-3
	20d	Present results of all sensitivity analyses conducted to assess the robustness of the synthesized results.	Page 11-15, Appendix C 1-3
Reporting biases	21	Present assessments of risk of bias due to missing results (arising from reporting biases) for each synthesis assessed.	page 15-16, Appendix D
Certainty of evidence	22	Present assessments of certainty (or confidence) in the body of evidence for each outcome assessed.	page 11-16
**DISCUSSION**			
Discussion	23a	Provide a general interpretation of the results in the context of other evidence.	Page 17-18
	23b	Discuss any limitations of the evidence included in the review.	Page 19
	23c	Discuss any limitations of the review processes used.	Page 19
	23d	Discuss implications of the results for practice, policy, and future research.	Page 20
**OTHER INFORMATION**			
Registration and protocol	24a	Provide registration information for the review, including register name and registration number, or state that the review was not registered.	Page 3
	24b	Indicate where the review protocol can be accessed, or state that a protocol was not prepared.	Page 3
	24c	Describe and explain any amendments to information provided at registration or in the protocol.	Page 3
Support	25	Describe sources of financial or non-financial support for the review, and the role of the funders or sponsors in the review.	Page 20
Competing interests	26	Declare any competing interests of review authors.	Page 20
Availability of	27	Report which of the following are publicly available and where they can be found: template data collection forms; data extracted from included	Page 20

Section and Topic	Item #	Checklist item	Location where item is reported
data, code and other materials		studies; data used for all analyses; analytic code; any other materials used in the review.	

From: Page MJ, McKenzie JE, Bossuyt PM, Boutron I, Hoffmann TC, Mulrow CD, et al. The PRISMA 2020 statement: an updated guideline for reporting systematic reviews. BMJ 2021;372:n71. doi: 10.1136/bmj.n71. This work is licensed under CC BY 4.0. To view a copy of this license, visit https://creativecommons.org/licenses/by/4.0/

Table S2- List of Excluded Studies, rationales for their exclusion, and stage of exclusion.

**Author**	**Rationales for exclusion**	**Stage**
1.		
Kwok, J.Y.Y.; Choi, E.P.H.; Wong, J.Y.H.; Lok, K.Y.W.; Ho, M.-H.; Fong, D.Y.T.; Kwan, J.C.Y.; Pang, S.Y.Y.; Auyeung, M. A Randomized Clinical Trial of Mindfulness Meditation versus Exercise in Parkinson's Disease during Social Unrest. *NPJ Parkinson's Disease* **2023**, *9*, 7, doi:10.1038/s41531-023-00452-w.	Study design	screening
2.		
Pitts, L.L.; Cox, A.; Morales, S.; Hannah, T. A Systematic Review and Meta-Analysis of Iowa Oral Performance Instrument Measures in Persons with Parkinson's Disease Compared to Healthy Adults. *Dysphagia* **2022**, *37*, 99–115, doi:10.1007/s00455-021-10254-y.	outcome	eligibility
3.		
Cruickshank, T.M.; Reyes, A.R.; Ziman, M.R. A Systematic Review and Meta-Analysis of Strength Training in Individuals With Multiple Sclerosis Or Parkinson Disease. *Medicine* **2015**, *94*, e411, doi:10.1097/MD.0000000000000411.	population	eligibility
4.		
Borges, L.; Fernandes, A.; Oliveira dos Passos, J.; Rego, Ia.O.; Campos, T. Action Observation for Upper Limb Rehabilitation after Stroke. *Cochrane Database of Systematic Reviews* **2022**, doi:10.1002/14651858.CD011887.pub3.	population	eligibility
5.		
Fernández del Olmo, M.Á.; Sánchez, J.A.; Morenill, L.; Gómez Varela, J.; Fernández-Lago, H.; Bello, O.; Santos García, D. Aerobic and Resistance Exercises in Parkinson's Disease: A Narrative Review. **2018**.	Publication type	screening
6.		
Salgado, S.; Williams, N.; Kotian, R.; Salgado, M. An Evidence-Based Exercise Regimen for Patients with Mild to Moderate Parkinson's Disease. *Brain sciences* **2013**, *3*, 87–100.	method	screening
7.		
Uphoff, E.; Ekers, D.; Robertson, L.; Dawson, S.; Sanger, E.; South, E.; Samaan, Z.; Richards, D.; Meader, N.; Churchill, R. Behavioural Activation Therapy for Depression in Adults. *Cochrane Database of Systematic Reviews* **2020**, doi:10.1002/14651858.CD013305.pub2.	population	eligibility
8.		
Luong Thanh, B.; Laopaiboon, M.; Koh, D.; Sakunkoo, P.; Moe, H. Behavioural Interventions to Promote Workers' Use of Respiratory Protective Equipment. *Cochrane Database of Systematic Reviews* **2016**, doi:10.1002/14651858.CD010157.pub2.	population	eligibility

9.		
Lenouvel, E.; Ullrich, P.; Siemens, W.; Dallmeier, D.; Denkinger, M.; Kienle, G.; Zijlstra, G.; Hauer, K.; Klöppel, S. Cognitive Behavioural Therapy (CBT) with and without Exercise to Reduce Fear of Falling in Older People Living in the Community. *Cochrane Database of Systematic Reviews* **2023**, doi:10.1002/14651858.CD014666.pub2.	population	eligibility
10.		
Ballesteros, J.; Moreno-Calvete, M.; Santos-Zorrozúa, B.; González-Fraile, E. Cognitive Behavioural Therapy plus Standard Care versus Standard Care for Persistent Aggressive Behaviour or Agitation in People with Schizophrenia. *Cochrane Database of Systematic Reviews* **2023**, doi:10.1002/14651858.CD013511.pub2.	population	eligibility
11.		
Li, H.; Lin, X.; Huang, R.; Huang, S.; Wu, X. Comparative Effects of Medication Combined with Twenty Rehabilitation Therapies: Core Outcomes in 8202 Parkinson's Patients. *NPJ Parkinson's disease* **2026**, doi:10.1038/s41531-026-01266-2.	Full text not available	screening
12.		
Harris, D.M.; Rantalainen, T.; Muthalib, M.; Johnson, L.; Duckham, R.L.; Smith, S.T.; Daly, R.M.; Teo, W.-P. Concurrent Exergaming and Transcranial Direct Current Stimulation to Improve Balance in People with Parkinson's Disease: Study Protocol for a Randomised Controlled Trial. *Trials* **2018**, *19*, 387, doi:10.1186/s13063-018-2773-6.	Study design	screening
13.		
Hendy, A.M.; Tillman, A.; Rantalainen, T.; Muthalib, M.; Johnson, L.; Kidgell, D.J.; Wundersitz, D.; Enticott, P.G.; Teo, W.-P. Concurrent Transcranial Direct Current Stimulation and Progressive Resistance Training in Parkinson's Disease: Study Protocol for a Randomised Controlled Trial. *Trials* **2016**, *17*, 326, doi:10.1186/s13063-016-1461-7.	Type of publication	screening
14.		
Li, J.A.; Loevaas, M.B.; Guan, C.; Goh, L.; Allen, N.E.; Mak, M.K.Y.; Lv, J.; Paul, S.S. Does Exercise Attenuate Disease Progression in People With Parkinson's Disease? A Systematic Review With Meta-Analyses. *Neurorehabil Neural Repair* **2023**, *37*, 328–352, doi:10.1177/15459683231172752.	outcome	eligibility
15.		
Thabrew, H.; Stasiak, K.; Hetrick, S.; Wong, S.; Huss, J.; Merry, S. E-Health Interventions for Anxiety and Depression in Children and Adolescents with Long-term Physical Conditions. *Cochrane Database of Systematic Reviews* **2018**, doi:10.1002/14651858.CD012489.pub2.	population	eligibility
16.		
Tian, J.; Kang, Y.; Liu, P.; Yu, H. Effect of Physical Activity on Depression in Patients with Parkinson's Disease: A Systematic Review and Meta-Analysis. *International Journal of Environmental Research and Public Health* **2022**, *19*, 6849, doi:10.3390/ijerph19116849.	outcome	eligibility
17.		

Yang, Y.; Wang, Y.; Gao, T.; Reyila, A.; Liu, J.; Liu, J.; Han, H. Effect of Physiotherapy Interventions on Motor Symptoms in People with Parkinson's Disease: A Systematic Review and Meta-Analysis. *Biological research for nursing* **2023**, *25*, 586–605.	Full text not available	screening
18.		
Gandhi, P.; Steele, C.M. Effectiveness of Interventions for Dysphagia in Parkinson Disease: A Systematic Review. *American Journal of Speech - Language Pathology (Online)* **2022**, *31*, 463–485, doi:10.1044/2021_AJSLP-21-00145.	outcome	eligibility
19.		
Danielle Pessoa Lima; Brito de Almeida, S.; Janine de Carvalho Bonfadini; Emmanuelle Silva Tavares Sobreira; Patrícia Gomes Damasceno; Antonio Brazil Viana Júnior; Madeleine Sales de Alencar; João Rafael Gomes de Luna; Barros Rodrigues, P.G.; de Sousa Pereira, I.; et al. Effects of a Power Strength Training Using Elastic Resistance Exercises on the Motor and Non-Motor Symptoms in Patients with Parkinson's Disease H&Y 1–3: Study Protocol for a Randomised Controlled Trial (PARK-BAND Study). *BMJ Open* **2020**, *10*, doi:10.1136/bmjopen-2020-039941.	Study design	screening
20.		
Guo, X.B.; Tang, L. Effects of Different Exercise Types on Balance Function in Healthy Older Adults and Parkinson's Patients: A Systematic Review. *Frontiers in aging neuroscience* **2024**, *16*, 1411584, doi:10.3389/fnagi.2024.1411584.	population	eligibility
21.		
de Almeida, F.O.; Santana, V.; Corcos, D.M.; Ugrinowitsch, C.; Silva-Batista, C. Effects of Endurance Training on Motor Signs of Parkinson's Disease: A Systematic Review and Meta-Analysis. *Sports Medicine* **2022**, *52*, 1789–1815, doi:10.1007/s40279-022-01650-x.	method	screening
22.		
Sun, G.; Ding, X.; Zheng, Z.; Ma, H. Effects of Exercise Interventions on Cognitive Function in Patients with Cognitive Dysfunction: An Umbrella Review of Meta-Analyses. *Frontiers in aging neuroscience* **2025**, *17*, 1553868, doi:10.3389/fnagi.2025.1553868.	population	eligibility
23.		
Yan, H.-L.; Li, M.-M. Effects of Exercise Modalities on Cognitive Function and Depression in Patients with Parkinson's Disease: A Systematic Review and Network Meta-Analysis. *Geriatric nursing (New York, N.Y.)* **2025**, *66*, 103683, doi:10.1016/j.gerinurse.2025.103683.	outcome	eligibility
24.		
Huang, S.; Zhang, L. Effects of Exercise Modality-Dose Combinations on Cognitive Function in Parkinson's Disease: A Bayesian Dose-Response Systematic Review and Meta-Analysis. *Archives of gerontology and geriatrics* **2025**, *139*, 105996, doi:10.1016/j.archger.2025.105996.	outcome	eligibility
25.		

Sherindan Ayessa Ferreira de Brito; Aline Alvim Scianni; Bruna Mara Franco Silveira; Elem Rodrigues Martins de Oliveira; Mateus, M.E.; Christina Danielli Coelho de Morais Faria Effects of High-Intensity Respiratory Muscle Training on Respiratory Muscle Strength in Individuals with Parkinson's Disease: Protocol of a Randomized Clinical Trial. *PLoS One* **2023**, *18*, doi:10.1371/journal.pone.0291051.	Study design	screening
26.		
Yu, J.; Wu, J.; Lu, J.; Wei, X.; Zheng, K.; Bowen, L.; Xiao, W.; Shi, Q.; Xiong, L.; Ren, Z. Efficacy of Virtual Reality Training on Motor Performance, Activity of Daily Living, and Quality of Life in Patients with Parkinson's Disease: An Umbrella Review Comprising Meta-Analyses of Randomized Controlled Trials. *Journal of Neuroengineering and Rehabilitation* **2023**, *20*, 1–11, doi:10.1186/s12984-023-01256-y.	method	screening
27.		
Yong-Hui, Z.; Hao-Yu, H.; Yuan-Chang, X.; Peng, C.; Hu, L.; Ya-Zhuo Kong; Yu-Ling, W.; Jia-Bao, G.; Bi, S.; Tie-Shan, L.; et al. Exercise for Neuropathic Pain: A Systematic Review and Expert Consensus. *Frontiers in Medicine* **2021**, *8*, 756940, doi:10.3389/fmed.2021.756940.	population	eligibility
28.		
Steiger, L.; Homann, C.N. Exercise Therapy in Parkinson's Disease–An Overview of Current Interventional Studies. *Physiotherapy Research and Reports* **2019**, *1*, 1–10.	Publication Type	screening
29.		
Kim, Y.; Lai, B.; Mehta, T.; Thirumalai, M.; Padalabalanarayanan, S.; Rimmer, J.H.; Motl, R.W. Exercise Training Guidelines for Multiple Sclerosis, Stroke, and Parkinson Disease: Rapid Review and Synthesis. *Am J Phys Med Rehabil* **2019**, *98*, 613–621, doi:10.1097/PHM.0000000000001174.	population	eligibility
30.		
Mitchell, A.K.; Bliss, R.R.; Church, F.C. Exercise, Neuroprotective Exerkines, and Parkinson's Disease: A Narrative Review. *Biomolecules* **2024**, *14*, 1241, doi:10.3390/biom14101241.	outcome	eligibility
31.		
Ge, Y.; Zhao, W.; Zhang, L.; Zhao, X.; Shu, X.; Li, J.; Qiao, L.; Liu, Y.; Wang, H. Home Physical Therapy versus Telerehabilitation in Improving Motor Function and Quality of Life in Parkinson's Disease: A Randomized Controlled Trial. *BMC Geriatrics* **2024**, *24*, 1–11, doi:10.1186/s12877-024-05529-6.	Study design	screening
32.		
Ramos, T.L.; de Sousa Fernandes, M.S.; da Silva Fidelis, D.E.; de Carvalho Martins, J.C.; Filho, E.M.A.A.; do Nascimento, H.R.; do Nascimento, I.R.; dos Santos, L.F.; de Souza, R.F. Impacts of High-Intensity Aerobic and Resistance Training on Functional Capacity of Patients with Parkinson's Disease: A Systematic Review Impactos Do Treinamento Aeróbico e Resistido de Alta Intensidade Na Capacidade Funcional de Pacientes Com Doença de Parkinson: Uma.	Full text not available	screening

33.		
Hazelton, C.; Thomson, K.; Todhunter-Brown, A.; Campbell, P.; Chung, C.; Dorris, L.; Gillespie, D.; Hunter, S.; McGill, K.; Nicolson, D.; et al. Interventions for Perceptual Disorders Following Stroke. *Cochrane Database of Systematic Reviews* **2022**, doi:10.1002/14651858.CD007039.pub3.	population	eligibility
34.		
Hayes, S.; Galvin, R.; Kennedy, C.; Finlayson, M.; McGuigan, C.; Walsh, C.; Coote, S. Interventions for Preventing Falls in People with Multiple Sclerosis. *Cochrane Database of Systematic Reviews* **2019**, doi:10.1002/14651858.CD012475.pub2.	population	eligibility
35.		
Tsoi, D.; Porwal, M.; Webster, A. Interventions for Smoking Cessation and Reduction in Individuals with Schizophrenia. *Cochrane Database of Systematic Reviews* **2013**, doi:10.1002/14651858.CD007253.pub3.	population	eligibility
36.		
Spiga, F.; Davies, A.; Tomlinson, E.; Moore, T.; Dawson, S.; Breheny, K.; Savović, J.; Gao, Y.; Phillips, S.; Hillier-Brown, F.; et al. Interventions to Prevent Obesity in Children Aged 5 to 11 Years Old. *Cochrane Database of Systematic Reviews* **2024**, doi:10.1002/14651858.CD015328.pub2.	population	eligibility
37.		
Handoll, H.; Cameron, I.; Mak, J.; Panagoda, C.; Finnegan, T. Multidisciplinary Rehabilitation for Older People with Hip Fractures. *Cochrane Database of Systematic Reviews* **2021**, doi:10.1002/14651858.CD007125.pub3.	population	eligibility
38.		
Mendes Oliveira, L.; de Araújo Júnior, H.C.; Renee Felipe, T.; Menezes Cruz, H.V.; Woloszin, G.; Civiero, M.; de Souza Pessoa, B.V.; Soares Macedo, M.T. NEUROPLASTICIDADE INDUZIDA PELO EXERCÍCIO FÍSICO NA DOENÇA DE PARKINSON. *Revista Brasileira de Prescrição e Fisiologia do Exercício* **2024**, *18*, 226–238.	outcome	eligibility
39.		
O'Connell, N.; Marston, L.; Spencer, S.; DeSouza, L.; Wand, B. Non-invasive Brain Stimulation Techniques for Chronic Pain. *Cochrane Database of Systematic Reviews* **2018**, doi:10.1002/14651858.CD008208.pub5.	population	eligibility
40.		
Hernández-Triana, D.; Páez-García Salomón; Mena Alexandre; Gimeno Mar; Soto-Leal, A.; Rodriguez-Oroz, M.C.; Borda, M.G. Parkinson's Disease and Frailty: A Two-Way Link Across Aging. *Journal of Clinical Medicine* **2025**, *15*, 63, doi:10.3390/jcm15010063.	outcome	eligibility
41.		

Luiza, V.; Chaves, L.; Silva, R.; Emmerick, I.; Chaves, G.; Fonseca de Araújo, S.; Moraes, E.; Oxman, A. Pharmaceutical Policies: Effects of Cap and Co-payment on Rational Use of Medicines. *Cochrane Database of Systematic Reviews* **2015**, doi:10.1002/14651858.CD007017.pub2.	population	eligibility
42.		
Mücke, M.; Mochamat, m; Cuhls, H.; Peuckmann-Post, V.; Minton, O.; Stone, P.; Radbruch, L. Pharmacological Treatments for Fatigue Associated with Palliative Care. *Cochrane Database of Systematic Reviews* **2015**, doi:10.1002/14651858.CD006788.pub3.	population	eligibility
43.		
Osborne, J.A.; Botkin, R.; Colon-Semenza, C.; DeAngelis, T.R.; Gallardo, O.G.; Kosakowski, H.; Martello, J.; Pradhan, S.; Rafferty, M.; Readinger, J.L.; et al. Physical Therapist Management of Parkinson Disease: A Clinical Practice Guideline From the American Physical Therapy Association. *PTJ: Physical Therapy & Rehabilitation Journal* **2022**, *102*, 1–36, doi:10.1093/ptj/pzab302.	Type of publication	screening
44.		
Cheng, F.; Yang, Y.; Chen, L.; Wu, Y.; Cheng, S.; Wang, R. Positive Effects of Specific Exercise and Novel Turning-Based Treadmill Training on Turning Performance in Individuals with Parkinson's Disease: A Randomized Controlled Trial. *Scientific Reports (Nature Publisher Group)* **2016**, *6*, 33242, doi:10.1038/srep33242.	Type of publication	screening
45.		
Kampling, H.; Baumeister, H.; Bengel, J.; Mittag, O. Prevention of Depression in Adults with Long-term Physical Conditions. *Cochrane Database of Systematic Reviews* **2021**, doi:10.1002/14651858.CD011246.pub2.	population	eligibility
46.		
Vieira De Moraes Filho, A.; Chaves, S.N.; Martins, W.R.; Tolentino, G.P.; Homem, R.; Landim De Farias, G.; Fischer, B.L.; Oliveira, J.A.; Pereira, S.K.A.; Vidal, S.E.; et al. Progressive Resistance Training Improves Bradykinesia, Motor Symptoms and Functional Performance in Patients with Parkinson's Disease. *CIA* **2020**, 87–95, doi:10.2147/CIA.S231359.	Type of publication	screening
47.		
Schipper, S.; Nigam, K.; Schmid, Y.; Piechotta, V.; Ljuslin, M.; Beaussant, Y.; Schwarzer, G.; Boehlke, C. Psychedelic-assisted Therapy for Treating Anxiety, Depression, and Existential Distress in People with Life-threatening Diseases. *Cochrane Database of Systematic Reviews* **2024**, doi:10.1002/14651858.CD015383.pub2.	population	eligibility
48.		
Drahota, A.; Udell, J.; Mackenzie, H.; Pugh, M. Psychological and Educational Interventions for Preventing Falls in Older People Living in the Community. *Cochrane Database of Systematic Reviews* **2024**, doi:10.1002/14651858.CD013480.pub2.	population	eligibility
49.		

Thabrew, H.; Stasiak, K.; Hetrick, S.; Donkin, L.; Huss, J.; Highlander, A.; Wong, S.; Merry, S. Psychological Therapies for Anxiety and Depression in Children and Adolescents with Long-term Physical Conditions. *Cochrane Database of Systematic Reviews* **2018**, doi:10.1002/14651858.CD012488.pub2.	population	eligibility
50.		
Comemale, É. Recommandation Pour l'Activité Physique En Neurologie Centrale : Quelle Posologie Pour Quels Effets ? Une Revue Systématique de La Littérature. *Kinésithérapie Revue* **2022**, *22*, 18–33, doi:10.1016/j.kine.2022.04.011.	population	eligibility
51.		
Gardoni, A.; Sarasso, E.; Agosta, F.; Filippi, M.; Corbetta, D. Rehabilitative Interventions for Impaired Handwriting in People with Parkinson's Disease: A Scoping Review. *Neurological Sciences* **2023**, *44*, 2667–2677, doi:10.1007/s10072-023-06752-6.	outcome	eligibility
52.		
Brown, R.; Cherian, K.; Jones, K.; Wickham, R.; Gomez, R.; Sahlem, G. Repetitive Transcranial Magnetic Stimulation for Post-traumatic Stress Disorder in Adults. *Cochrane Database of Systematic Reviews* **2024**, doi:10.1002/14651858.CD015040.pub2.	population	eligibility
53.		
Biebl, J.T.; Azqueta-Gavaldon, M.; Wania, C.; Zettl, O.; Woiczinski, M.; Bauer, L.; Storz, C.; Bötzel, K.; Kraft, E.; Hélio Teive Resistance Training Combined with Balance or Gait Training for Patients with Parkinson's Disease: A Randomized Controlled Pilot Study. *Parkinson's Disease* **2022**, *2022*, doi:10.1155/2022/9574516.	Study design	screening
54.		
Peek, A.L.; Stevens, M.L. Resistance Training for People with Parkinson's Disease (PEDro Synthesis). *Br J Sports Med* **2016**, *50*, 1158, doi:10.1136/bjsports-2016-096311.	Full text not available	screening
55.		
Navas-Garrido, I.; Martín-Núñez, J.; Raya-Benítez, J.; Granados-Santiago, M.; Navas-Otero Alba; López-López, L.; Valenza, M.C. Respiratory Muscle Strength Training in Parkinson's Disease—A Systematic Review and Meta-Analysis. *Healthcare* **2025**, *13*, 1214, doi:10.3390/healthcare13101214.	outcome	eligibility
56.		
Bhalsing, K.; Abbas, M.; S. Tan, L. Role of Physical Activity in Parkinson's Disease. *Ann Indian Acad Neurol* **2018**, *21*, 242, doi:10.4103/aian.AIAN_169_18.	Type of publication	screening
57.		
Jønsson, A.B.; Krogh, S.; Laursen, H.S.; Aagaard, P.; Kasch, H.; Nielsen, J.F. Safety and Efficacy of Blood Flow Restriction Exercise in Individuals with Neurological Disorders: A Systematic Review. *Scandinavian Journal of Medicine & Science in Sports (John Wiley & Sons, Inc.)* **2024**, *34*, 1–18, doi:10.1111/sms.14561.	population	eligibility

58.		
Slinger, C.; Mehdi, S.; Milan, S.; Dodd, S.; Matthews, J.; Vyas, A.; Marsden, P. Speech and Language Therapy for Management of Chronic Cough. *Cochrane Database of Systematic Reviews* **2019**, doi:10.1002/14651858.CD013067.pub2.	population	eligibility
59.		
Gosler, M.; Testroote, M.; Morrenhof, J.; Janzing, H. Surgical versus Non-surgical Interventions for Treating Humeral Shaft Fractures in Adults. *Cochrane Database of Systematic Reviews* **2012**, doi:10.1002/14651858.CD008832.pub2.	population	eligibility
60.		
Sousa, C.; Sales, M.; Rosa, T.; Lewis, J.; Andrade, R.; Simões, H. The Antioxidant Effect of Exercise: A Systematic Review and Meta-Analysis. *Sports Medicine* **2017**, *47*, 277–293, doi:10.1007/s40279-016-0566-1.	population	eligibility
61.		
Smith, M.; Barker, R.; Williams, G.; Carr, J.; Gunnarsson, R. The Effect of Exercise on High-Level Mobility in Individuals with Neurodegenerative Disease: A Systematic Literature Review. *Physiotherapy* **2020**, *106*, 174–193, doi:10.1016/j.physio.2019.04.003.	population	eligibility
62.		
Bajwah, S.; Oluyase, A.; Yi, D.; Gao, W.; Evans, C.; Grande, G.; Todd, C.; Costantini, M.; Murtagh, F.; Higginson, I. The Effectiveness and Cost-effectiveness of Hospital-based Specialist Palliative Care for Adults with Advanced Illness and Their Caregivers. *Cochrane Database of Systematic Reviews* **2020**, doi:10.1002/14651858.CD012780.pub2.	population	eligibility
63.		
Tang, L.; Fang, Y.; Yin, J. The Effects of Exercise Interventions on Parkinson's Disease: A Bayesian Network Meta-Analysis. *Journal of clinical neuroscience : official journal of the Neurosurgical Society of Australasia* **2019**, *70*, 47–54, doi:10.1016/j.jocn.2019.08.092.	Full text not available	screening
64.		
Wen, X.; Liu, Z.; Liu, X.; Peng, Y.; Liu, H. The Effects of Physiotherapy Treatments on Dysphagia in Parkinson's Disease: A Systematic Review of Randomized Controlled Trials. *Brain research bulletin* **2022**, *188*, 59–66, doi:10.1016/j.brainresbull.2022.07.016.	outcome	eligibility
65.		
Hortobágyi, T.; Vetrovsky, T.; Balbim, G.M.; Sorte Silva, N.C.B.; Manca, A.; Deriu, F.; Kolmos, M.; Kruuse, C.; Liu-Ambrose, T.; Radák, Z.; et al. The Impact of Aerobic and Resistance Training Intensity on Markers of Neuroplasticity in Health and Disease. *Ageing research reviews* **2022**, *80*, 101698, doi:10.1016/j.arr.2022.101698.	population	eligibility
66.		
Majed Awad Alanazi The Role of Physical Activity in Adjunctive Nursing Management of Neuro-Degenerative Diseases among Older Adults: A Systematic Review of Interventional Studies. *Life* **2024**, *14*, 597, doi:10.3390/life14050597.	outcome	eligibility
67.		

van Hooren, M.R.A.; Baijens, L.W.J.; Voskuilen, S.; Oosterloo, M.; Kremer, B. Treatment Effects for Dysphagia in Parkinson's Disease: A Systematic Review. *Parkinsonism & related disorders* **2014**, *20*, 800–807, doi:10.1016/j.parkreldis.2014.03.026.	outcome	eligibility
68.		
López-Liria, R.; Parra-Egeda, J.; Vega-Ramírez, F.A.; Aguilar-Parra, J.M.; Trigueros-Ramos, R.; Morales-Gázquez, M.J.; Rocamora-Pérez, P. Treatment of Dysphagia in Parkinson's Disease: A Systematic Review. *International Journal of Environmental Research and Public Health* **2020**, *17*, 4104, doi:10.3390/ijerph17114104.	outcome	eligibility
69.		
Dockx, K.; Bekkers, E.; Van den Bergh, V.; Ginis, P.; Rochester, L.; Hausdorff, J.; Mirelman, A.; Nieuwboer, A. Virtual Reality for Rehabilitation in Parkinson's Disease. *Cochrane Database of Systematic Reviews* **2016**, doi:10.1002/14651858.CD010760.pub2.	population	eligibility

Table S3- Literature Search

MyEBSCO –

SU (parkinson's disease) AND SU (resistance training or strength training or weight lifting or resistance exercise or strength exercise) AND (systematic review or meta analysis or literature review)

ProQuest-

title(parkinson') AND abstract(progressive resistance training OR strength training OR resistance training) AND (systematic literature reviews) AND stype.exact("Scholarly Journals") AND (at.exact("Evidence Based Healthcare" OR "Literature Review" OR "Review") AND PEER(yes))

Limited by:
Full text

Source type:

Scholarly Journals

All dates

Cochran-

- **S1:** [mh "Parkinson Disease"]
- **S2:** (Parkinson*):ti,ab,kw .
- **S3:** #1 OR #2
- **S4:** [mh Exercise]
- **S5:** ("resistance training" OR "strength training" OR "physical exercise"):ti,ab,kw
- **S6:** #4 OR #5
- **S7:** #3 AND #6

PubMed-

(Parkinson Disease[MeSH Terms] OR Parkinson*[Title/Abstract])

AND

(Resistance Training[MeSH Terms] OR "Strength Training"[Title/Abstract] OR "Progressive Resistance"[Title/Abstract])

AND

(Systematic Review[Filter] OR Meta-Analysis[Filter])

Google Scholar-

"Parkinson Disease" AND "Resistance Training" OR "Strength Training" OR "Progressive Resistance Training" AND "Systematic Review" OR "Meta-Analysis"

Table S4- An Explanation of the Coding System via the study of Cherup et al[1]

Framework	Group	Type	Volume	Intensity	Frequency	Progressive Overload	Specificity	Variation
**ACSM**	S	2	2	2	2			
	P	2	2	2	2			
**Supplementary**	S	0	2	2	2			
	P	0	2	2	2			
**Principles of Progressive Overload**	S					2	0	2
	P					2	0	2
Cherup et al. [1]<br><br>**Outcomes: Neuromuscular deficits**<br>S+<br>P+<br>**Functional performance**<br>S-<br>P-	S v P	Pneumatic Resistance Machines	3x10 reps	70% 1RM (S) or 50% 1RM (P). Tempo: Explosive con/2–3s ecc for p. 2-3 sec con /ecc for s	2x/wk; 12 wk	5–10% increase	Leg press, seated row, calf raise, lat pulldown, hip abduction, hip adduction, biceps curl, triceps pushdown, shoulder press, and chest press	Two-week familiarization. Modification due to manifestation of patient-specific PD symptoms.

ACSM-American College of Sports Medicine. S- strength. P- Power. WK- Week. Con- concentric. Ecc- Eccentric
+ effect - no effect

To illustrate the application of the three-tiered evaluative framework, the study by Cherup et al.[1], serves as a representative case for how interventions were systematically coded. This example highlights how a protocol can demonstrate high adherence to foundational standards while showing lower adherence to specialized clinical criteria.

ACSM FITT-VP –

- **Type-** The protocols utilized resistance machines, which align with ACSM safety and delivery standards for this population, earning **2 points (high adherence)**.
- **Intensity-** The protocols consisted of intensity of 50% RM (P) and 70% RM (S), which met the criteria of the ACSM that stated a range of 30-60% 1RM for beginners, and 60-80% 1 RM for advanced exercisers**.** This criterion was coded with **2 points (high adherence).**
- **Volume and Frequency:** The interventions were conducted twice weekly for 12 weeks, consisting of 3 sets of 10 repetitions per exercise. This structure met the criteria for **2 points (high adherence)** in both categories.

Integrated Guidelines

- **Type**- Since the protocols utilized resistance machines, and the Integrated Guidelines on prescription of balance and functional activities, this criterion was coded with **0 points (no adherence)**.
- **Volume, Frequency, and Intensity**- The supplementary applies recommendations for healthy adults to people with PD, while the authors implemented modifications to account for PD symptoms while maintaining recommended loads, earning **2 points (high adherence)**.

Principles of Progressive Overload

- **Progressive Overload and Variation**- The study included a familiarization phase, specific modifications, and systematic increases in external loads. Consequently, these criteria were coded with **2 points (high adherence)**.

- **Specificity**- Although strength increased, the protocols did not include functional tasks adequate to the demands of the functional outcome measures (such as balance, sit-to-stand, and walking). Therefore, specificity was coded with **0 points (no adherence)** .

A specialist in sports science, who is the first author, systematically evaluated each intervention using a 3-point adherence scale: 2 (high adherence), 1 (uncertain/partial adherence), and 0 (non- adherence) [11]. These ratings were based on the specific recommendations established within each of the three frameworks detailed in Tables 1 through 3. Adherence patterns across the included literature were synthesized using descriptive statistics. Following the methodology established by Cui et al. [11], a threshold of ≥70% was utilized to categorize an intervention as demonstrating "high adherence," while scores <70% were classified as "low or uncertain adherence". Assessing adherence to the ACSM's recommendations and using this type of coding system also appears in other studies designated for various populations[[2–9]].

Table S5- Adherence of Clinical Studies to FITT ACSM Recommendations

Authors, Year	Groups	Effect	Type	Volume	Intensity	Frequency	Total	Proportion
Corcos et al. [55]	PRT<br>Modified Fitness Count (MFC)	**UPDRS-III**: PRT +	2	2	1	2	7/8	87.5%
Prodoehl et al. [65]	PRT<br>MFC	**Functional performance**<br>PRT +<br>mFC +	2	2	1	2	7/8	87.5%
Santos et al. [66]	PRT<br>Balance	**Postural control** Balance+	1	2	0	2	5/8	62.5%
Dibble et al. [57]	PRT ecc<br>General Fitness	**Muscle hypertrophy**<br>**Muscle force**<br>**Mobility**<br>ecc PRT +	1	0	2	2	5/8	62.5%
Dibble et al. [58]	PRT ecc<br>General Fitness	**Muscle force production**<br>**Bradykinesia**<br>**QoL**<br>ecc PRT +	1	0	2	2	5/8	62.5%
Dibble et al. [39]	PRT eccentric<br>PRT concentric	**Body Structure/function**<br>ecc PRT +<br>con PRT +<br>(Both have little effect on participation).	1	0	2	2	5/8	62.50%
Vieira De Moraes Filho et al. [71]	PRT	**Bradykinesia functional performance**<br>PRT +	2	2	2	2	8/8	100%

	Passive control group							
Ferreira et al. [45]	PRT<br>Passive control group	**Anxiety and QoL**<br>PRT +	1	2	2	2	7/8	87.5%
Schilling et al. [67]	PRT<br>Standard Care	**Maximum strength** PRT +<br>**6 MWT**<br>**ABC**	2	1	1	2	6/8	75%
De Lima et al. [72]	PRT<br>Passive control group	**Depressive symptoms, QoL, Functional Performances**<br>PRT+	2	2	2	2	8	100%
Helgerad et al. [40]	Body weight + maximal PRT<br>Body weight + low /medium PRT	**Force-generating capacity**<br>**Efferent neural drive functional performance**<br>PRT +	1	1	1	0	3/8	37.50%
Hirsch et al. [60]	High-intensity PRT with balance<br>Only balance	**Strength**<br>**Balance**<br>PRT with balance+	2	2	0	2	6/8	75%
Li et al. [62]	Tai chi	**Falls**	2	2	1	2	7/8	87.50%

	PRT<br>Stretching	Tai Chi>PRT> stretching						
Schlenstedt et al. [68]	PRT<br>Balance	**postural control**<br>(Small, non-significant)<br>PRT+<br>Balance+	2	1	1	2	6/8	75%
Schlenstedt et al. [41]	PRT<br>Balance	**FOG**<br>PRT-<br>Balance-	2	1	1	2	6/8	75%
Strand et al. [42]	PRT H<br>PRT F	**functional capacity Balance**<br>strength+<br>power +	H-2<br>F-2	H-2<br>F-2	H-2<br>F-2	H-2<br>F-2	8/8	100%
Silva Batista et al. [43]	PRTI PRT<br>Passive control group	**Strength**<br>PRT+<br>PRTI+<br>**mobility, motor signs, and QoL**<br>PRTI+	PRT-2<br>PRTI-2	PRT-2<br>PRTI-2	PRT-2<br>PRTI-2	PRT-2<br>PRTI-2	8/8	100%
Silva Batista et al. [44]	PRTI<br>PRT<br>Passive control group	**Balance**<br>**fear of falling**<br>PRTI+<br>**Other balance outcomes**<br>PRTI>PRT	PRT-2<br>PRTI-2	PRT-2<br>PRTI-2	PRT-2<br>PRTI-2	PRT-2<br>PRTI-2	8/8	100%

Silva Batista et al. [44]	PRTI<br>PRT<br>Passive control group	**Neuromuscular adaptations** (to improve mobility)<br>PRTI +<br>**Other neuromuscular outcomes**<br>PRTI>PRT	PRT-2<br>PRTI-2	PRT-2<br>PRTI-2	PRT-2<br>PRTI-2	PRT-2<br>PRTI-2	8/8	100%
Silva Batista et al. [47]	PRTI<br>Traditional motor rehabilitation	**Clinical improvement**<br>**Brain plasticity** (in freezers)<br>PRTI+	2	2	2	2	8/8	100%
Morris et al. [48]	PRT+ falls prevention education<br>Movement strategy falls prevention education<br>Passive control group	**Rate of falls**<br>RCFP with PRT+<br>RCFP with MST+	2	2	1	2	7/8	87.50%
Leal et al. [61]	Low-volume PRT Passive control group	**Physical capacity** PRT +	1	1	2	2	6/8	75%
Allen et al. [53]	PRT+ balance<br>Passive control group	**fall risk**<br>**strength**<br>**FoG**<br>**STS**	2	N/A	2	2	6/8	75%

		PRT with Balance+						
Cherap et al. [49]	PRT S<br>PRT P	**Neuromuscular deficits**<br>S+<br>P+<br>**Functional performance**<br>S-<br>P-	PRT S-2<br>PRT-2	PRT S-2<br>PRT-2	PRT S-2<br>PRT-2	PRT S-2<br>PRT-2	8/8	100%
Shulman et al. [69]	PRT+ stretching<br>High-intensity treadmill<br>Low-intensity treadmill	**Gait speed**<br>Treadmill >PRT& Stretching<br>**Muscle strength**<br>PRT& Stretching+	2	1	1	2	6/8	75%
Paul et al. [64]	PRT power<br>Control group (RT sham)	**Power**<br>**strength**<br>P+<br>**Balance**<br>**Mobility**<br>**Falls**<br>P?	1	2	2	2	7/8	87.5%
Alessandro Carvalho et al. [54]	PRT<br>Aerobic	**Disease symptoms**<br>**Functional capacity**<br>S +<br>Aerobic +	2	2	2	2	8/8	100%
Ni et al. [63]	Designed yoga	**Physical performance**<br>Yoga+	2	2	2	2	8/8	100%

	P	P+						
Marie Domonceau & Didier Maxuet [56]	PRT<br>Aerobic	**Compliance with training specificities**<br>PRT+<br>Aerobic+<br>(did not translate to better mobility or QoL)	2	2	2	2	8/8	100%
Alves et al. [50]	PRT<br>Passive control group	**Respiratory muscle strength QoL**<br>S+	2	1	2	2	7/8	87.5%
Viera -Yano et al. [46]	PRTI<br>TMR	**Gait speed**<br>**Stride length**<br>PRTI+<br>TMR+<br>**Gait (automaticity, attentional set shifting)**<br>PRTI>TMR	PRT-1<br>PRTI-1	PRT-2<br>PRTI-2	PRT-2<br>PRTI-2	PRT-2<br>PRTI-2	7/8	87.5%
Hass et al. [59]	PRT<br>Control group- current lifestyle without initiation of strength or balance	**Gait Initiation Performance**<br>PRT +	2	1	1	2	6/8	75%
Shen & Mak [51]	PRT	**Speed** (immediately)<br>PRT+	2	0	0	2	4/8	50%

	Balance with augmented feedback and gait training	Balance+<br>**balance confidence** (12-month carryover)<br>Balance+						
Shen & Mak [52]	PRT<br>Balance with augmented feedback and gait training	**Falls**<br>technology-assisted balance and gait training+	2	0	0	2	4/8	50%

FITT- Frequency, Intensity, Time, and Type. ACSM-American College of Sports Medicine. Volume: the total amount of exercise. PRT- Progressive Resistance Training. MFC- Modified Fitness Count. ROM- Range of Motion. QoL- Quality of Life. H- Hypertrophy. F- Functional. FOG- Freezing of Gate. PRTI- Progressive Resistance Training with Instability. TTV- Total Training Volume. S- strength. P- Power. TMR - Traditional Motor Rehabilitation. Con- concentric. Ecc- Eccentric

+ effect ± small effect >greater effect - no effect ? not definite

*A proportion of ≥70% designated high adherence, and <70% designated low or uncertain adherence. *Score Key: 2 = High Adherence, 1 = Uncertain Adherence, 0 = No Adherence.

Table S6- Adherence of Clinical Studies to ACSM Guidelines

**Authors, Year**	**Groups**	**Effect**	**Type**	**Volume**	**Intensity**	**Frequency**	**Total**	**Proportion**
Corcos et al. [55]	PRT<br>Modified Fitness Count (MFC)	**UPDRS-III**: PRT +	0	2	2	2	6/8	75%
Prodoehl et al. [65]	PRT<br>MFC	**Functional performance**<br>PRT +<br>mFC +	0	2	2	2	6/8	75%
Santos et al. [66]	PRT<br>Balance	**Postural control** Balance+	0	2	0	2	4/8	50%
Dibble et al.[57]	PRT eccentric<br><br>General Fitness	**Muscle hypertrophy**<br>**Muscle force**<br>**Mobility**<br>ecc PRT +	1	0	2	2	5/8	62.5%
Dibble et al. [58]	PRT eccentric<br>PRT concentric	**Body Structure/function**<br>ecc PRT +<br>con PRT +<br>(Both have little effect on participation).	1	0	2	2	5/8	62.5%
Dibble et al. [39]	PRT eccentric<br>PRT concentric	**Body Structure/function**<br>ecc PRT +<br>con PRT +<br>(Both have little effect on participation).	1	0	2	2	5/8	62.50%

Vieira De Moraes Filho et al. [71]	PRT<br>Passive control group	**Bradykinesia functional performance**<br>PRT +	0	2	2	2	6/8	75%
Ferreira et al. [45]	PRT<br>Passive control group	**Anxiety and QoL**<br>PRT +	1	2	2	2	7/8	87.5%
Schilling et al. [67]	PRT<br>Standard Care	**Maximum strength**<br>PRT +<br>**6 MWT**<br>**ABC**	0	1	2	2	5/8	62.5%
De Lima et al. [72]	PRT<br>Passive control group	**Depressive symptoms, QoL, Functional Performances**<br>PRT+	2	2	2	2	8	100%
Helgerad et al. [40]	Body weight + maximal PRT<br>Body weight + low /medium PRT	**Force-generating capacity**<br>**Efferent neural drive functional performance**<br>PRT +	2	1	2	1	6/8	75%
Hirsch et al. [60]	High-intensity PRT with balance<br>Only balance	**Strength**<br>**Balance**<br>PRT with balance+	2	2	2	2	8/8	100%
Li et al. [62]	Tai chi<br>PRT	**Falls**<br>Tai Chi>PRT> stretching	2	2	0	2	6/8	75%

	Stretching							
Schlenstedt et al. [68]	PRT<br>Balance	**postural control**<br>(Small, non-significant)<br>PRT+<br>Balance+	1	0	1	2	4	50%
Schlenstedt et al. [41]	PRT<br>Balance	**FOG**<br>PRT-<br>Balance-	1	0	1	2	4	50%
Strand et al. [42]	PRT functional<br>PRT hypertrophy	**functional capacity Balance**<br>strength+<br>power +	H- 1<br>F-2	H-2<br>F-2	H-2<br>F-1	H-2<br>F-2	7/8	87.5%
Silva Batista et al. [43]	PRT instability<br>PRT<br>Passive control group	**Strength**<br>PRT+<br>PRTI+<br>**mobility, motor signs, and QoL**<br>PRTI+	P-1<br>PI-2	P-2<br>PI-2	P-2<br>PI-2	P-2<br>PI-2	7/8<br>8/8	P-87.50%<br>PI-100%
Silva Batista et al. [44]	PRT instability<br>PRT<br>Passive control group	**Balance**<br>**fear of falling**<br>PRTI+<br>**Other balance outcomes**<br>PRTI>PRT	P-1<br>PI-2	P-2<br>PI-2	P-2<br>PI-2	P-2<br>PI-2	7/8<br>8/8	P-87.50%<br>PI-100%
Silva Batista et al. [44]	PRT instability<br>PRT<br>Passive control group	**Neuromuscular adaptations** (to improve mobility)<br>PRTI +<br>**Other neuromuscular outcomes**	P-1<br>PI-2	P-2<br>PI-2	P-2<br>PI-2	P-2<br>PI-2	7/8<br>8/8	P-87.50%<br>PI-100%

		PRTI>PRT						
Silva Batista et al. [47]	PRT instability<br>Traditional motor rehabilitation	**Clinical improvement**<br>**Brain plasticity** (in freezers)<br>PRTI+	2	2	2	2	8	100%
Morris et al. [48]	PRT+ falls prevention education<br>Movement strategy falls prevention education<br>Passive control group	**Rate of falls**<br>RCFP with PRT+<br>RCFP with MST+	2	2	1	2	6	75%
Leal et al. [61]	Low-volume PRT Passive control group	**Physical capacity** PRT +	1	2	2	2	7	87.5%
Allen et al. [53]	PRT+ balance<br>Passive control group	**fall risk**<br>**strength**<br>**FoG**<br>**STS**<br>PRT with Balance+	2	N/A	2	2	6	75%
Cherap et al. [49]	PRT strength<br>PRT power	**Neuromuscular deficits**<br>S+<br>P+<br>**Functional performance**<br>S-	S-0<br>P-0	S-2<br>P-2	S-2<br>P-2	S-2<br>P-2	S-6<br>P-6	S- 75%<br>P-75%

		P-						
Shulman et al. [69]	PRT+ stretching<br>High-intensity treadmill<br>Low-intensity treadmill	**Gait speed**<br>Treadmill >PRT& Stretching<br>**Muscle strength**<br>PRT& Stretching+	1	0	1	2	4	50%
Paul et al. [64]	PRT power<br>Control group (RT sham)	**Power strength**<br>P+<br>**Balance**<br>**Mobility**<br>**Falls**<br>P?	1	2	2	2	7	87.5%
Alessandro Carvalho et al. [54]	PRT<br>Aerobic	**Disease symptoms**<br>**Functional capacity**<br>S +<br>Aerobic +	0	2	2	2	6	75%
Ni et al. [63]	Designed yoga<br>Power	**Physical performance**<br>Yoga+<br>P+	0	2	2	2	6	75%
Marie Domonceau & Didier Maxuet [56]	PRT<br>Aerobic	**Compliance with training specificities**<br>PRT+<br>Aerobic+<br>(did not translate to better mobility or QoL)	0	2	2	2	6	75%

Alves et al. [50]	PRT<br>Passive control group	**Respiratory muscle strength QoL**<br>S+	1	1	2	2	6	75%
Viera -Yano et al. [46]	PRT<br>Instability<br>Traditional Motor Rehabilitation (TMR)	**Gait speed**<br>**Stride length**<br>PRTI+<br>TMR+<br>**Gait (automaticity, attentional set shifting)**<br>PRTI>TMR	P-1<br>PI-2	P-1<br>PI-2	P-2<br>PI-2	P-2<br>PI-2	P-6<br>PI-8	P- 75%<br>PI-100%
Hass et al. [59]	PRT<br>Control group-current lifestyle without initiation of strength or balance	**Gait Initiation Performance**<br>PRT +	0	1	2	2	5	62.5%
Shen & Mak [51]	PRT<br>Balance with augmented feedback and gait training	**Speed** (immediately)<br>PRT+<br>Balance+<br>**balance confidence** (12-month carryover)<br>Balance+	1	0	1	2	4	50%
Shen & Mak [52]	PRT<br>Balance with augmented	**Falls**<br>technology-assisted balance and gait training+	1	0	1	2	4	50%

	feedback and gait training							

FITT- Frequency, Intensity, Time, and Type. ACSM-American College of Sports Medicine. Volume: the total amount of exercise. PRT- Progressive Resistance Training. MFC- Modified Fitness Count. ROM- Range of Motion. QoL- Quality of Life. H- Hypertrophy. F- Functional. FOG- Freezing of Gate. PRTI- Progressive Resistance Training with Instability. TTV- Total Training Volume. S- strength. P- Power. TMR - Traditional Motor Rehabilitation. Con- concentric. Ecc- Eccentric

+ effect ± small effect >greater effect - no effect ? not definite

*A proportion of ≥70% designated high adherence, and <70% designated low or uncertain adherence. *Score Key: 2 = High Adherence, 1 = Uncertain Adherence, 0 = No Adherence.

Table S7-adherence of Clinical Studies to the Principles of Progression

**Authors, Year**	**Groups**	**Effect**	**Load**	**specific**	**variation**	**Total Score**	**Adherence %**
Corcos et al. [55]	PRT Modified Fitness Count (MFC)	**UPDRS-III**: PRT +	2	0	2	4	**66.6%**
Prodoehl et al. [65]	PRT MFC	**Functional performance** PRT + mFC +	2	0	2	4	**66.6%**
Santos et al. [66]	PRT Balance	**Postural control** Balance+	0	0	0	0	**0**
Dibble et al. [57]	PRT ecc General Fitness	**Muscle hypertrophy Muscle force Mobility** ecc PRT +	2	1	1	4	**66.6%**
Dibble et al. [58]	PRT ecc General Fitness	**Muscle force production**	2	1	1	4	**66.6%**

		**Bradykinesia**<br>**QoL**<br>ecc PRT +					
Dibble et al. [39]	PRT eccentric PRT concentric	**Body Structure/function** ecc PRT + con PRT + (Both have little effect on participation).	2	1	1	4	**66.6%**
Vieira De Moraes Fhilo et al. [71]	PRT Passive control group	**Bradykinesia functional performance** PRT +	2	0	2	4	**66.6%**
Ferreira et al. [45]	PRT Passive control group	**Anxiety and QoL** PRT +	2	1	2	5	**83.3%**
Schilling et al. [67]	PRT Standard Care	**Maximum strength** PRT + **6 MWT**	2	0	2	4	**66.6%**

		**ABC**					
De Lima et al. [72]	**Depressive symptoms, QoL, Functional Performances** PRT+	PRT Passive control group	2	1	2	5	**83.3%**
Helgerad et al. [40]	Body weight + maximal PRT Body weight + low /medium PRT	**Force-generating capacity Efferent neural drive functional performance** PRT +	2	2	1	5	**83.3%**
Hirsch et al. [60]	High-intensity PRT with balance Only balance	**Strength Balance** PRT with balance+	2	2	2	6	**100%**
Li et al. [62]	Tai chi PRT Stretching	**Falls** Tai Chi>PRT> stretching	0	2	1	3	**50%**

Schlenstedt et al. [68]	PRT<br>Balance	**postural control** (Small, non-significant)<br>PRT+<br>Balance+	1	0	2	2	**50%**
Schlenstedt et al. [41]	PRT<br>Balance	**FOG**<br>PRT-<br>Balance-	1	0	2	2	**50%**
Strand et al. [42]	PRT functional<br>PRT hypertrophy	**functional capacity**<br>**Balance**<br>strength+<br>power +	H- 2<br>F-1	H-1<br>F-2	H-2<br>F-2	5	**83.3%**
Silva Batista et al. [43]	PRT instability<br>PRT<br>Passive control group	**Strength**<br>PRT+<br>PRTI+<br>**mobility, motor signs, and QoL**<br>PRTI+	P-2<br>PI-2	P-1<br>PI-2	P-2<br>PI-2	5<br>6	**83.3%**<br>**100%**
Silva Batista et al. [44]	PRT instability<br>PRT<br>Passive control group	**Balance**<br>**fear of falling**<br>PRTI+	P-2<br>PI-2	P-1<br>PI-2	P-2<br>PI-2	5<br>6	**83.3%**<br>**100%**

		**Other balance outcomes**<br><br>PRTI>PRT					
Silva Batista et al. [44]	PRT instability<br>PRT<br>Passive control group	**Neuromuscular adaptations** (to improve mobility)<br>PRTI +<br>**Other neuromuscular outcomes**<br>PRTI>PRT	P-2<br>PI-2	P-1<br>PI-2	P-2<br>PI-2	5<br>6	**83.3%**<br>**100%**
Silva Batista et al. [47]	PRT instability<br>Traditional motor rehabilitation	**Clinical improvement**<br>**Brain plasticity** (in freezers)<br>PRTI+	2	2	2	6	**100%**
Morris et al. [48]	PRT+ falls prevention education	**Rate of falls**<br>RCFP with PRT+	0	2	2	4	**66.6%**

	Movement strategy for falls prevention education<br>Passive control group	RCFP with MST+					
Leal et al. [61]	PRT<br>Passive control group	**Physical capacity**<br>PRT +	2	1	2	5	**83.3%**
Allen et al. [53]	PRT+ balance<br>Passive control group	**fall risk**<br>**strength**<br>**FoG**<br>**STS**<br>PRT with Balance+	0	1	2	3	**50%**
Cherap et al. [49]	PRT strength<br>PRT power	**Neuromuscular deficits**<br>S+<br>P+<br>**Functional performance**	S-2<br>P-2	S-0<br>P-0	S-2<br>P-2	S-4<br>P-4	**66.6%**

		S-<br>P-					
Shulman et al. [69]	PRT+ stretching<br>High-intensity treadmill<br>Low-intensity treadmill	**Gait speed**<br>Treadmill > PRT& Stretching<br>**Muscle strength**<br>PRT& Stretching+	0	1	0	1	**16.6%**
Paul et al. [64]	PRT power<br>Control group (sham)	**Power strength**<br>P+<br>**Balance**<br>**Mobility**<br>**Falls**<br>P?	2	1	2	5	**83.3%**
Alessandro Carvalho et al. [54]	PRT<br>Aerobic	**Disease symptoms**<br>**Functional capacity**<br>S +<br>Aerobic +	2	0	2	4	**66.6%**
Ni et al. [63]	Designed yoga<br>Power	**Physical performance**<br>Yoga+<br>P+	2	0	2	4	**66.6%**

Marie Domonceau & Didier Maxuet [56]	PRT<br>Aerobic	**Compliance with training specificities**<br>PRT+<br>Aerobic+<br>(did not translate to better mobility or QoL)	2	0	2	4	**66.6%**
Alves et al. [50]	PRT<br>Passive control group	**Respiratory muscle strength**<br>**QoL**<br>S+	2	1	2	5	**83.3%**
Viera -Yano et al. [46]	PRT<br>Instability<br>Traditional Motor Rehabilitation (TMR)	**Gait speed**<br>**Stride length**<br>PRTI+<br>TMR+<br>**Gait (automaticity, attentional set shifting)**<br>PRTI>TMR	P-2<br>PI-2	P-2<br>PI-2	P-1<br>PI-2	P-5<br>PI-6	**P-83.3%**<br>**PI-100%**

Hass et al. [59]	PRT Control group- current lifestyle without initiation of strength or balance	**Gait Initiation Performance** PRT +	1	0	2	3	**50%**
Shen & Mak [51]	PRT Balance with augmented feedback and gait training	**Speed** (immediately) PRT+ Balance+ **balance confidence** (12-month carryover) Balance+	0	1	0	1	**16.6%**
Shen & Mak [52]	PRT Balance with augmented feedback and gait training	**Falls** technology-assisted balance and gait training+	0	1	0	1	**16.6%**

PRT- Progressive Resistance Training. MFC- Modified Fitness Count. ROM- Range of Motion. QoL- Quality of Life. H- Hypertrophy. F- Functional. FOG- Freezing of Gate. PRTI- Progressive Resistance Training with

Instability. TTV- Total Training Volume. S- strength. P- Power. TMR - Traditional Motor Rehabilitation. Con-concentric. Ecc- Eccentric

+ effect ± small effect >greater effect - no effect ? not definite

*A proportion of ≥70% designated high adherence, and <70% designated low or uncertain adherence. *Score Key: 2 = High Adherence, 1 = Uncertain Adherence, 0 = No Adherence.

Table S8- Details on Interventions from Systematic Reviews

Author(s) & Year	Design (N)	Population (Age; H&Y)	Protocol (Freq; Dur)	Comparison	Modalities Analyzed	Primary Outcomes	Key Conclusion
Gollan et al. [1]	18 RCTs	18+; H&Y 1–4	1–3d/wk; 7wk–2yr	PRT vs. Passive/Active	PRT, Balance, Endurance, Aqua	Strength, Motor, FoG, QoL, Depression	PRT effective<br>No differences between interventions
Ernst et al. [2]	156 RCTs	59–74; H&Y 1–4	~12wk	Ex vs. Passive/Active	PRT, Dance, Aqua-Based Gait/Balance/Functional, Multi Domain, Mind Body, Endurance, LSVT BIG, Flexibility, Gaming,	Motor signs, QoL	PRT might affect Motor Signs<br>PRT has un certain effect on QoL.<br>No differences between most interventions
Paolucci et al. [3]	8 Reviews	18+; H&Y 0–3	2–3d/wk; 10wk	PRT vs. Passive/Active	PRT, Treadmill, Balance, Stretch	Strength, Gait, Mobility, QoL	PRT should be combined with balance training to preserve fitness and postural control.
Lima et al. [4]	4 Trials	57–76; H&Y 1.8–2.5	2–3d/wk; 8–24wk	PRT vsPassive/Active	PRT, Walking, Balance	Strength, Gait (speed/6MWT), TUG	PRT is effective for walking capacity, but carryover to all

							physical measures is inconsistent.
X. Yang & Wang [5]	14 RCTs	59–75; NR	2–3d/wk; 1.5–24mo	PRT vs. Passive/Active	PRT, Balance, Stretching	FoG, Strength, QoL, Gait	PRT has positive effects on FOG, strength, and overall QoL
Karpodini et al. [6]	49 Studies	40–107; H&Y 1–5	1 sess–24mo	Ex vs. Passive/Active	PRT, Rhythmic Cueing, Dance	UPDRS-III, TUG, Stride, QoL	Rhythmic cueing, dance, and PRT all positively affect motor manifestations.
X. Li et al. [7]	31 RCTs	59–80; H&Y 1–4	1–3d/wk; 6wk–24mo	PRT vs. Passive/Active	PRT, Yoga, Treadmill, Balance, MFC, stretching, regular exercise	Strength, QoL, Gait, Balance	PRT improves strength and QoL; balance effects require further study.
Tillman et al. [8]	7 RCTs	63–70; H&Y 1–3	2–3d/wk; 8–24wk	PRT vs. Passive/Active	PRT, Treadmill, Standard Care	strength, Gait speed, Balance	PRT effect on strength. Combine PRT with task-specific training for functional gains.
Y. Yang et al. [9]	250 RCTs	50+; H&Y 2.35 (avg)	~3d/wk; ~12wk	Ex vs. Passive	20+ types (Dance, BWS_TT, PRT, Yoga, etc.)	Motor, Balance, FoG, Strength	Power, BWS_TT, dance Yoga, and PRT are highly effective depending on the

							outcome of interest.
Song et al. [10]	15 RCTs	50–70; H&Y 1–4	1–5d/wk; 7–24wk	Ex vs. Passive/Active	Aerobic vs. Resistance	UPDRS-III, Gait velocity, Balance	Aerobic superior for motor/gait; PRT superior for balance (Mini-BESTest).
Álvarez-Bueno [11]	56 Trials	58–78; H&Y 1–4	1–5d/wk; 2wk–3yr	Ex vs. . Passive/Active	Endurance, RT, Dance, Stretching, Balance, Dance, Alternative exercises Body weight supported, combined exercises	Motor symptoms (UPDRS)	Sensorimotor training (including endurance and PRT) is most effective for motor management.
De Almeida [12]	4 RCTs	~70; H&Y 1–3	2–3d/wk; 12wk	PRT vs. . Passive/Active	Power, Aerobic, Strength, Yoga	Mobility (TUG), Balance, Gait	Power training did not show superiority; more high-volume/intensity studies needed.
Zhou et al. [13]	20 RCTs	59–73; H&Y 1–3	2–4d/wk; 4–26wk	Ex vs. . Passive/Active	Aerobic & PRT (var. intensity)	UPDRS-III, 6MWT, TUG, QoL, 10MWT	Short-period high-intensity PRT is a recommended complementary therapy.

Chung et al. [14]	7 RCTs	50–77; H&Y 1–4	2–3d/wk; 8–24wk	PRT vs. . Passive/Active	PRT	Strength, Balance, Motor signs, Gait QoL	Moderate intensity PRT (8–10 weeks) yields significant gains in strength and balance.
Palheta De Lima [15]	10 Trials	Older Adults; H&Y 2	NR; 8–12wk	Ex vs. . Passive/Active	PRT & Balance Training	Balance, Agility, FoG, UPDRS	PRT is effective for motor activities; specific balance training is needed for postural stability.
Zhang et al. [16]	159 RCTs	58–77; H&Y 1–4	1–10d/wk; 2–96wk	Ex vs. Passive/Active	20+ types (PRT, Pilates, Aquatic)	TUG, Stride length, Stride cadence, 6MWT	Benefits vary by type: PRT Pilates, BWS_TT, preferable for gait performance and TUG.
Brienesse [17]	5 Studies	50–82; H&Y 0–3	1–3d/wk; 8–12wk	PRT vs. Passive/Active	PRT, standard care	Strength, Endurance, Functional	PRT offers strength and functional gains; prescription needs a more robust study.
Roeder et al. [18]	9 Trials	~68; H&Y 1–3	2–3d/wk; 6wk–6mo	PRT vs. Passive/Active	PRT alone or combined	Muscle strength (Knee Ext/Flex)	PRT is effective combining RT with other modes is most effective.

Hao et al. [19]	60 RCTs	58–74; H&Y 1–4	1–7d/wk; 4–24wk	Ex vs. Passive/Active	Dance, Yoga, VR, RT, Aquatic, Treadmill, cycling, walking, tai chi	BBS, UPDRS, TUG	Dance, yoga, VR, and RT offer better advantages for motor function.
Lamotte et al. [20]	6 RCTs	40–85; H&Y 1–4	2–3d/wk; 8wk–24mo	PRT vs. Passive/Active	PRT	UPDRS, physical performance (Gait, Balance), QoL, cognition,	PRT improves strength and motor signs; carryover to physical function is probable.
Chamberlain-Carter [21]	11 RCTs	40–90; H&Y 1–3	1–3d/wk; 8wk–18mo	PRT vs. Active	ST	Strength, Falls, QoL	ST improves strength with carryover effects on falls and quality of life.
Zhaoli et al. [22]	13 RCTs	40–70; NR	NR; 8–104wk	PRT vs. Passive/Active	RT (Core/Functional)	BBS, TUG, Stride length, walking speed	Improves balance and mobility, and walking ability; no significant benefit for stride length or walking speed.
Gamborg et al. [23]	40 Studies	~65; H&Y 1–4	NR	PD vs. Healthy Control	RT, Power	Strength, Power, MDS-UPDRS, RFD, Functional capacity, Balance, Gait, TUG	Muscle function is impaired in PD; strength is associated with

							functional capacity.
Wang et al. [24]	81 Trials	~65; H&Y 2.35	1–7d/wk; 4–96wk	Ex vs. Passive/Active	PRT, Dance, Yoga, Sensory, Balance, Gait, BWS, Tai chi, Mixed exercises	Motor symptom Optimal dose (by METs), MCID	Non-linear dose-response; 1300 MET-min/week is optimal for symptom relief. Dance affects at a dose of 850 MET, PRT, BWS, and Sensory affects at lower doses
Gamborg et al. [25]	33 RCTs	65; H&Y 1–4.4	~3d/wk; 4–104wk	Ex vs. Passive/Active	PRT, Endurance, OITM,	Strength, Cardiorespiratory Fitness, TUG, UPDRS, QoL, 6MW	PRT, endurance, and OITM are safe and beneficial adjunct strategies. PRT and endurance show a solid effect.
Padilha et al. [26]	139 Reviews	18–90; H&Y 1–4	1–7d/wk; 1–104wk	Ex vs. Passive/Active	Aerobic, PRT, Combined, sensory	Motor, QoL, Balance, Walking, Fatigue, Functional, Non-motor outcomes,	Combined exercises may yield positive effects on both motor and non-motor outcomes. Physical exercise

							affects motor outcomes.
Saltychev et al. [27]	12 RCTs	59–71; NR	2–3d/wk; 1.5–24mo	PRT vs. Passive/Active	PRT	Aerobic, Speed, Maximal oxygen consumption, TUG, 6MWT	Currently, there is no evidence to support the superiority of PRT over other forms of physical training.
Ramazzina et al. [28]	13 RCTs	59–76; H&Y 1.5–3	1–5d/wk; 3–104wk	PRT vs. Active	PRT (Weights, Bands, etc.)	Strength, Balance, Gait, FoG, UPDRS, Physical performance, QoL	PRT against external resistance is well tolerated and improves physical/QoL parameters.
Tonkin et al. [29]	5 RCTs	67; H&Y 2.2	1–3d/wk; 10–52wk	Ex vs. Active/Passive	Only Dual task: Balance and Gait, Mixed Treadmill, Dance and Movement, PRT, and BW Functional, Tango	Non-motor (UPDRS Part 1)	Tango and treadmill training are most effective for non-motor experiences.
Xie et al. [30]	54 RCTs	53–74; H&Y 3.1	1–7d/wk; 2–48wk	Ex vs. Passive/Active	PRT, Aerobic, Mind-Body, Balance, and Gait, Multicomponent, Sensory,	Walking velocity	Significant improvements with PRT, Aerobic Mind Body, Multicomponent; optimal PRT

							dose at 750 MET-min/week. No exercise achieved MCID
Tambosco et al. [31]	5 reviews 31 RCTs	61-74; H&Y ~2	3d/wk; 1wk–16mo	Ex vs. Passive/Active	Aerobic, Strength	VO2 Max, Strength, UPDRS, Gait, Balance, QoL, Falls, Non-motor outcomes	Aerobic and PRT training improve physical abilities; intensity and medication are the key.
Mendes da Costa et al., [32]	11 RCTs	~ >60; H&Y 2.	2-3 d\wk 8-24 wk;	Ex vs. Passive/Active	PRT, Aerobic, Balance, yoga, Proprioceptive	UPDRS, TUG, PDQ-39	PRT and balance (in combination or alone) improve static balance, dynamic balance, and agility. For motor symptoms, any exercise modality, as long as it is periodized and systematized.
Hvingelby et al., [33]	148 RCTs	N\A, H&Y 1-5	N\A, 2wk- 2 years	Ex vs. Passive/Active	20+ types (PRT, Aerobic, Proprioceptive, Robot Assisted Gait)	Dynamic gait, Fitness, Balance, FoG, UPDRS	Aquatic therapy and dual-task training are effective for dynamic gait; treadmill

							training improves motor scores. No single intervention is superior for all symptoms.
Braz De Oliveira et al., [34]	10 RCTs	~66, H&Y 1–3	2x/wk; 8–24 wk	Ex vs. Passive/Active	PRT	Strength, Fitness, Balance, Flexibility, Gait, Mobility, QoL	PRT improves upper limb strength, balance, and gait, but shows no significant effect on QoL.
Mao et al., [35]	55 RCTs	60–77; Disease duration ~6.8 yrs	3x/wk;4 wk- 12 months	Ex vs. Active	Dance, Aerobic, PRT, Virtual Reality, Aquatic, Mind-body, Sensory, Exoskeletal,	Balance, Cognition, Emotional Function, QoL	Dance and Exoskeleton are most effective for balance. PRT and Aquatic are best for emotion. Mind-body is best for cognition. PRT is best for QoL
Uhrband et al., [36]	15 RCTs	59-68; H&Y ~1.2	N\A; 4-104 WK	Ex vs. Passive/Active	PRT, Aerobic, OITM	Strength, Cardio-respiratory fitness, Walking, UPDRS, QoL	Strong evidence that PRT improves strength, and Aerobic improves fitness.

							Beneficial effects of PRT, Aerobic, and OITM on balance, walking, and UPDRS
Tong et al., [37]	64 RCTs	56-75; Disease duration 3-17 yrs	1-6\ wk 4-24 wk	Ex vs. Active	Routine, Aerobic, Postural Control, Mind-body, Tai Chi, Sensory, PRT	Gait stabilization, Fall risk, Postural control	Mind-body exercise is most effective for gait stabilization; Routine physical training is best for fall risk; Postural control training is best for postural control
Yuan et al., [38]	73 RCTs	>50; H&Y <4	1-7/wk; 2–48 weeks;	Ex vs. Passive/Active	Aquatic, Mixed, Sensory, PRT, Mind-body, Dance	TUG	Aquatic exercise is the most effective (optimal 1500 METs-min/week). PRT shows a U-shaped dose-response (optimal 610 METs). Mind-body is effective at lower doses

RCT- Randomized Controlled Trials, PRT- Progressive Resistance Training. QoL- Quality of Life. FoG- Freezing of Gate. WK- Week. Yrs- Years. N- Number. H&Y- Hoehn & Yahar (stage of disease). PD- Parkinson's disease.
MCID- Minimum Clinically Important Difference, BWS- Body weight support exercise, OITM- Other intensive exercise modalities, Ex- Exercise, MET- Metabolic Equivalent, RFD- Rate of Force Development, TUG- Timed Up and Go, 6MW- 6 Minute Walk, BBS- Berg Balance Scale, Mini-BESTest- Mini Balance Evaluation Systems Test, PDQ -39- Parkinson's Disease questionary, UPDRS- Unified Parkinson Disease Rating Scale.

Table S9- Details on PRT outcome measures from Systematic Reviews

**Author(s) & Year**	**Strength**	**Motor Signs (UPDRS)**	**Functional Mobility (TUG, 6MWT, etc.)**	**Gait (Velocity, Stride)**	**Balance**	**QoL**	**FOG**
Gollan et al. [1]	Significant	Significant	No Sig	No Sig	No Sig	Significant	Significant
Ernst et al. [2]	+ Effect	+ Effect	Significant	Significant	Significant	+ Effect	No Sig
Paolucci et al. [3]	Significant	Significant	Significant	Significant	Significant	Significant	N/A
Lima et al. [4]	+ Effect	N/A	No Sig	Significant	No Sig	N/A	N/A
X. Yang & Wang [5]	Significant	N/A	No Sig	No Sig	No Sig	Significant	Significant
Karpodini et al. [6]	Significant	Significant	Significant	Significant	N/A	Significant	N/A
X. Li et al. [7]	Significant	N/A	Significant	Significant	Significant	Significant	Significant

Tillman et al. [8]	Significant	N/A	N/A	No Sig	No Sig	N/A	N/A
Y. Yang et al. [9]	Significant	Significant	No Sig	No Sig	No Sig	N/A	Significant
Song et al. [10]	N/A	Significant	No Sig	Significant	Significant	N/A	N/A
Álvarez-Bueno [11]	N/A	Effective	N/A	N/A	N/A	N/A	N/A
De Almeida [12]	N/A	N/A	No Sig	No Sig	No Sig	N/A	N/A
Zhou et al. [13]	N/A	Significant	Significant	Significant	N/A	Significant	N/A
Chung et al. [14]	Significant	Significant	No Sig	No Sig	Significant	No Sig	N/A
Palheta De Lima [15]	N/A	N/A	N/A	N/A	Significant	N/A	N/A
Zhang et al. [16]	N/A	N/A	Significant	Significant	N/A	N/A	N/A
Brienesse [17]	Significant	N/A	Significant	N/A	N/A	N/A	N/A
Roeder et al. [18]	Significant	N/A	N/A	N/A	N/A	N/A	N/A
Hao et al. [19]	N/A	No Sig	Significant	N/A	No Sig	N/A	N/A
Lamotte et al. [20]	Significant	Significant	+ Effect	+ Effect	+ Effect	Uncertain	N/A
Chamberlain-Carter [21]	N/A	N/A	N/A	N/A	N/A	+ Effect	N/A

Zhaoli et al. [22]	N/A	N/A	Significant	No Sig	Significant	N/A	N/A
Gamborg et al. [23]	Significant	Significant	Negative association	No to a strong association	No to a strong association	N/A	N/A
Wang et al. [24]	N/A	N/A	N/A	N/A	N/A	N/A	N/A
Gamborg et al. [25]	+ Effect	+ Effect	+ Effect	N/A	N/A	+ Effect	N/A
Padilha et al. [26]	N/A	+ Effect	+ Effect	Significant	+ Effect	+ Effect	N/A
Saltychev et al. [27]	N/A	N/A	No Sig	No Sig	N/A	N/A	N/A
Ramazzina et al. [28]	+ Effect	+ Effect	+ Effect	+ Effect	+ Effect	+ Effect	N/A
Tonkin et al. [29]	N/A	N/A	N/A	N/A	N/A	N/A	N/A
Xie et al. [30]	N/A	N/A	N/A	+ Effect	N/A	N/A	N/A
Tambosco et al. [31]	+ Effect	+ Effect	N/A	+ Effect	+ Effect	+ Effect	N/A
Mendes da Costa et al., [32]	N/A	+ Effect	No Sig	N/A	Significant	Significant	N/A
Hvingelby et al., [33]	N/A	No Sig	Significant (Fitness) / No Sig (Dynamic)	No Sig	No Sig	N/A	No Sig

Braz De Oliveira et al., [34]	Sig (Upper) / No Sig (Lower)	N/A	No Sig	Significant	Significant	No Sig	N/A
Mao et al., [35]	N/A	N/A	N/A	N/A	Significant (Low effect)	Significant	N/A
Uhrband et al., [36]	Significant	+ Effect (Inconsistent)	No Sig	+ Effect (Inconsistent)	+ Effect (Inconsistent)	+ Effect (Inconsistent)	N/A
Tong et al., [37]	N/A	N/A	N/A	Significant (Stabilization)	Significant (Control)	N/A	N/A
Yuan et al., [38]	N/A	N/A	Significant	N/A	N/A	N/A	N/A

TUG- Timed Up and Go, 6MW- 6 Minute Walk, QoL- Quality of Life, FoG- Freezing of Gait, UPDRS- Unified Parkinson Disease Rating Scale. **Sig**: Significant Effect reported compared to controls. **No Sig**: No significant difference compared to controls. **+** effect: Positive trend but not reported as significant. **N/A**: Outcome not specifically analyzed or reported for PRT in this review.

Table S10 - frequency table of the study's citation count and justifications for exclusion

Primary Study	Frequency	Exclusion
**Dibble et al.** [39]	**4**	
**Helgerud et al.** [40]	**1**	
**Schlenstedt et al.** [41]	**1**	
**Strand et al.** [42]	**1**	
**Silva Batista et al.** [43]	**4**	
**Silva Batista et al** [44]	**3**	
**Ferreira et al.** [45]	**7**	
**Viera -Yano et al.** [46]	**1**	
**Silva Batista et al.** [47]	**2**	

**Morris et al.** [48]	**7**	
**Cherup et al.** [49]	**3**	
**Alves et al.** [50]	**1**	
**Shen & Mak** [51]	**3**	
**Shen & Mak** [52]	**3**	
**Allen et al.,** [53]	**9**	
**Carvalho et al.,** [54]	**10**	
**Corcos et al.,** [55]	**10**	
**Domonceau et al.,** [56]	**5**	
**Dibble et al.,** [57]	**10**	
**Dibble et al.,** [58]	**3**	
**Hass et al., 2007**	**3**	With a Creatin supplement
**Hass et al.,**[59]	**13**	

**Hirsch et al.,** [60]	**11**	
**Leal et al.,** [61]	**4**	
**Li et al.,** [62]	**8**	
**Ni et al.,** [63]	**8**	
**Paul et al.,** [64]	**12**	
**Prodoehl et al.,** [65]	**4**	
**Santos et al.,** [66]	**10**	
**Schilling et al.,** [67]	**14**	
**Schlenstedt et al.,** [68]	**5**	
**Shulman et al.,** [69]	**10**	
**Silva-Batista et al.,** [70]	**5**	
**Tang et al., 2019**	**4**	Not in English
**Vieira de Moraes Filho et al.,**[71]	**7**	

**De lima 2019**[72]	**4**	
**Kanegusuku et al., 2017**	**3**	Not relevant outcome- cardiovascular
**Kwok et al., 2019**	**3**	Not relevant outcome- anxiety and depression
**Bloomer et al., 2008**	**3**	Not relevant outcome- oxidative stress
**Harvey et al., 2019**	**2**	Control group- wait list
**Mateos-Toset et al., 2016**	**1**	Method- 1 session
**Toole et al., 2000**	**1**	Method- pre and post

Table S11- Details of the Clinical Studies

Appendix D –					
**Authors, Year**	**N + PD Stage (H&Y)**	**Protocol & Duration**	**Intervention Details (Volume, Intensity, Tempo, Equipment, Cues, Progression)**	**Control Group Details**	**Key Conclusions**
Corcos et al. [55]	48 participants; H&Y 1.9–2.3	2x/wk; 24 months	Exercises: single & multi joint 3x8 reps (starting at 1 set). Intensity: 30–40% (upper) to 50–60% (lower) 1RM. Tempo: 6–9s per rep (2–3s con, 2–3s pause, 3–4s ecc). Progression: Alternated every 8 wks with strength + speed (70–80% 1RM); load increased by 5%.	Active: mFC; stretches, balance, breathing, non-progressive strengthening.	**UPDRS-III**: PRT +
Prodoehl et al. [65]	48 participants; H&Y 1.9–2.3	2x/wk; 24 months	Same PRT as [55]	Active: mFC; stretches, balance, breathing, and non-progressive strengthening.	**Functional performance** PRT + mFC +
Santos et al. [66]	40 participants; H&Y 1.5–3	2x/wk; 12 wk	2x10 reps; Intensity: Low load (1–2 kg). Exercises: Single joint. Fatigue: Designed to avoid	Active: Balance training (postural stability limits, sensory	**Postural control** Balance+

			accumulating fatigue in PD.	integration, coordination).	
Dibble et al. [39]	41 participants; H&Y 1–4	2x/wk; 12 wk	Eccentric: 15 min lower extremity ecc ergometry. Intensity: Target RPE 13. Progression: Load adjusted to maintain target RPE.	Active: Extremity con ergometry, General fitness (concentric leg training, cycle/treadmill, balance, flexibility).	**Body Structure/function** ecc PRT + con PRT + (Both have little effect on participation).
Vieira De Moraes Fhilo et al. [71]	40 participants; H&Y 1–3	2x/wk; 9 wk	2x10–12 reps to fatigue. Exercises: single & multi joints. Devices: weight machines	Passive: Control group attending lectures.	**Bradykinesia functional performance** PRT +
Ferreira et al. [45]	35 participants; H&Y 1–3	2x/wk; 6 mo	2x8–12 submaximal reps. exercises: single & multi joints (deadlift, unilateral row). **The author stated-weights adjusted in accordance with ACSM guidelines**	Passive: No-exercise control.	**Anxiety and QoL** PRT +
Dibble et al. [57]	19 participants;	3x/wk; 12 wk	Upper extremity: 3 sets: 12-15 reps: Devices: free weights, and Machines.	Active: General fitness (exercises with 3x 12/15 of 60-	**Muscle hypertrophy Muscle force Mobility**

	H&Y 1–3		Lower extremity: ecc ergometry. Started 3 min in the first week, and increased to 30 min in the last week. Intensity: Target RPE 13. Progression: intensity increased progressively across sessions. In addition to ecc, endurance (cycle/treadmill), balance, and flexibility.	70% 1 RM for upper and lower extremities, cycle/treadmill, balance, flexibility).	ecc PRT +
Dibble et al.[58]	19 participants; H&Y 1–3	3x/wk; 12 wk	Same PRT as [57]	Same PRT as [57]	**Muscle force production** **Bradykinesia** **QoL** ecc PRT +
Schilling et al. [67]	15 participants; H&Y 1.5–2.5	2x/wk; 8 wk	3x5–8 reps. Intensity: 2 sets of 8 reps and last set 5-8 reps; increase of 5-10% from a maximal effort to volitional fatigue. Exercises: single & multi joint (leg press, seated leg curl, and calf press). Tempo: Maximal con force, slow ecc	Active: standard care.	**Maximum strength** PRT + **6 MWT** **ABC**
De Lima et al. [72]	33 particip	2x/wk; 20 wk	2x8-12 reps	Passive: Standard	**Depressive symptoms,**

	ants; H&Y 1–3		Progression- between 2% and 10%<br>Exercises: single & multi joint.Free weight and machines<br>The author states- in accordance with ACSM guidelines	pharmacological treatment control.	**QoL, Functional Performances**<br>PRT+
Helgerad et al. [40]	22 participants; H&Y 1.3–3.3	5x/wk; 4 wk	Maximal Strength Training (MST): 4x4 reps at ~90% 1RM. Tempo: Maximal conc force; 0.5s pause; slow ecc. Progression: Load +5kg (leg) or +2.5kg (chest) if >4 reps possible.	Active: Conventional rehab (50% 1RM) + Nordic walking/stretching/lectures.	**Force-generating capacity**<br>**Efferent neural drive**<br>**functional performance**<br>PRT +
Hirsch et al. [60]	15 participants; H&Y 1.3–2.5	3x/wk; 10 wk	4 repetitions. Intensity: 80% of 4RM. Exercises: single joint. Tempo- 6-9 sec per repetition Progression: High-intensity resistance combined with balance.	Active: Only balance training.	**Strength**<br>**Balance**<br>PRT with balance+
Li et al. [62]	195 participants; H&Y 1–4	2x/wk; 24 wk	1–3 sets; 10–15 reps. Intensity: Weighted vests (initially 1% BW, progressed to 5%); ankle weights (0.45kg to 1.36kg). Progression: Load increased every 5th week	Active: Tai Chi vs. Stretching.	**Falls**<br>Tai Chi>PRT> stretching

			until 5% of body weight was achieved.		
Schlenstedt et al. [68]	40 participants; H&Y 2.5–3	2x/wk; 7 wk	3x15–20 reps to volitional fatigue. Exercises: single & multi joint. Progression: Once >20 reps reached, resistance (weights/bands) increased to maintain 15–20 rep range.	Active: Balance training (stance/gait with sensory manipulation).	**postural control** (Small, non-significant) PRT+ Balance+
Schlenstedt et al. [41]	20 participants; H&Y 2.4–3.4	2x/wk; 7 wk	Same PRT as [68]	Active: Balance training.	**FOG** PRT- Balance-
Strand et al. [42]	35 participants; H&Y 1–3	3x/wk; 12 wk	Strength/Power: 3x8 at 80% 1RM (Strength) or 3x6 at 50% 1RM (Power). Tempo: Fast concentric for Power. Progression: Compared to Hypertrophy or Functional circuits.	Active: PRT method comparison (Functional circuit vs. Hypertrophy group).	**functional capacity** **Balance** strength+ power +
Silva Batista et al. [43]	39 participants; H&Y 2–3	2x/wk; 12 wk	2–4 sets; 6–12 reps maximum. Exercises: (Half squat, chest/leg press). Progression: Sets/reps increased monthly. Instability: Unstable devices (Swiss ball, BOSU, etc.). For the instability	PRT vs. Passive: Educational lectures and Bingo games.	**Strength** PRT+ PRTI+ **mobility, motor signs, and QoL** PRTI+

			group, the degree of instability of the exercises also increased		
Silva Batista et al. [44]	39 participants; H&Y 2–3	2x/wk; 12 wk	Same instability protocol [43]	Passive: Educational lectures and Bingo games.	**Balance fear of falling** PRTI+ **Other balance outcomes** PRTI>PRT
Silva Batista et al. [44]	39 participants; H&Y 2–3	2x/wk; 12 wk	Same instability protocol [43]	Passive: Educational lectures and Bingo.	**Neuromuscular adaptations** (to improve mobility) PRTI + **Other neuromuscular outcomes** PRTI>PRT
Silva Batista et al. [47]	32 participants (freezers); H&Y 2.8–3.6	3x/wk; 12 wk	Adaptive RT with instability (ARTI): 7 free-weight exercises (squat, lunge, reverse fly, etc.). Intensity: Load adjusted for assigned repetitions. Progression: Increased motor complexity.	Active: TMR (group stretching, gait, balance).	**Clinical improvement Brain plasticity** (in freezers) PRTI+
Morris et al. [48]	210 participants;	2x/wk; 8 wk	1–3 sets; up to 15 reps. Intensity: RPE ≈ 5. Progression: Weight +2%	Active: Movement Strategy	**Rate of falls** RCFP with PRT+

	H&Y 1–4		BW or reps/sets increased once RPE dropped. Devices: Weighted vests, Thera-Band. supervision of the therapist, family member, carer, or independently at home.	Training (visual/verbal cues, mental rehearsal) vs. Life Skills.	RCFP with MST+
Leal et al. [61]	54 participants; H&Y 1–3	2x/wk; 6 mo	2x8–12 reps. Intensity: RPE 7–8. Progression: Load +2–10% when 12 reps reached in 2 consecutive sessions. Devices: Free weights and machines. Tempo: tendency for concentric muscle failure and co-contraction, rhythm reduction, apnea and RPE score 7–8(hard). One exercise specialist with 5 patients.	Passive: Control group receiving pharmacological treatment only.	**Physical capacity** PRT +
Allen et al. [53]	48 participants (at risk of falls)	3x/wk; 6 mo	Home-based warm-up + strengthening. Intensity: RPE 15. Progression: Weighted vests starting at 2% BW. Monthly therapist class for monitoring. Participants who experienced FOG also	Passive: Usual care.	**fall risk**<br>**strength**<br>**FoG**<br>**STS**<br>PRT with Balance+

			performed cueing strategies.		
Cherap et al. [49]	35 participants; H&Y 1–3	2x/wk; 12 wk	3x10 reps. Intensity: 70% 1RM (S) or 50% 1RM (P). Tempo: Explosive concentric/2–3s eccentric for P. Progression: 5–10% increase.	Active: S vs. P	**Neuromuscular deficits**<br>S+<br>P+<br>**Functional performance**<br>S-<br>P-
Shulman et al. [69]	67 participants; H&Y 2–3	3x/wk; 3 mo	2x10 reps on 3 machines. Intensity: Weight increased as tolerated. Exercises: single & multi joint Combined with stretching.	Active: High-intensity treadmill (70–80% HRR) vs. Low-intensity treadmill (40–50% HRR).	**Gait speed**<br>Treadmill >PRT & Stretching<br>**Muscle strength**<br>PRT& Stretching+
Paul et al. [64]	40 participants; H&Y 1.9–2	2x/wk; 12 wk	3x8 reps. Intensity: 40% (set 1) to 60% (set 3) 1RM. Tempo: "As fast as possible" con. Progression: 1RM +5% when 10 reps possible in set 3.	Active: Low-intensity home exercise (sham) (trunk/leg flexors) with intentionally insufficient loads.	**Power strength**<br>P+<br>**Balance Mobility Falls**<br>P?
Alessandro Carvalho et al. [54]	22 participants;	2x/wk; 12 wk	2x8–12 reps. Intensity: 70–80% 1RM. Tempo: 2–3s phases. Progression:	Active: Aerobic Training (treadmill at	**Disease symptoms**

	H&Y 1–3		Constant adjustment to maintain max reps. exercises: single & multi joint	60% VO2max) vs. Physiotherapy (no overload).	**Functional capacity**<br>S +<br>Aerobic +
Ni et al. [63]	37 participants; H&Y 1–3	2x/wk; 12 wk	3 circuits; 10–12 reps. Intensity: Optimal load. Progression: Load +5% weekly or when power output stagnates. Devices: Pneumatic machines. Cues: Individualized training.	Active: Yoga vs. Monthly health education class.	**Physical performance**<br>Yoga+<br>P+
Marie Domonceau & Didier Maxuet [56]	46 participants; H&Y 1.5–2	2–3x/wk; 12 wk	2–3 sets. Intensity: 50–60% (wks 1–5) to 80–90% 1RM (wks 6–12). Tempo: con fast; ecc slowly. Fatigue: RPE Borg Scale. exercises: single & multi joint. Devices- weight machines	Passive: Standard care group.	**Compliance with training specificities**<br>PRT+<br>Aerobic+<br>(did not translate to better mobility or QoL)
Alves et al. [50]	28 participants; H&Y 1–2	2x/wk; 16 wk	2x8–12 reps. Intensity: 60–80% 1RM. Tempo: 2s con, 2s ecc. Progression: Weight +2–10% when 12 reps possible. Fatigue/Cues: RPE 7–8; cues to avoid apnea. exercises: single & multi joint (deadlift, unilateral rowing).	Passive: Standard pharmacological treatment control.	**Respiratory muscle strength QoL**<br>S+

			Devices: weight machines. **The author states- in accordance with ACSM guidelines**		
Viera - Yano et al. [46]	32 participants; H&Y 3.1	3x/wk; 12 wk	2–4 sets; 6–12 reps. Intensity: Systematic load increase (5–10%). Devices: free weights + unstable discs/BOSU. Progression: Increased motor complexity.	Active: TMR (stretching, gait, balance, weight shifts) with systematic weight increase.	**Gait speed Stride length** PRTI+ TMR+ **Gait (automaticity, attentional set shifting)** PRTI>TMR
Hass et al. [59]	18 participants; H&Y 2.3	2x/wk; 10 wk	2x12–20 reps to fatigue. Intensity: 70% 1RM (knee ext/flex); other exercises +10% from orientation. Fatigue: Maximal effort encouraged. Exercises: single & multi joint.	Passive: Non-contact control (current lifestyle).	**Gait Initiation Performance** PRT +
Shen & Mak [51]	45 participants; H&Y 2–3	3–5x/wk; 12 wk	2x15 reps. Intensity: 60% 1RM. Progression: 1RM reassessed every 2 wks. Home-based: Step/walking with 0.5–1.5kg ankle sandbags. Fatigue: Supervised in lab.	Active: Computerized dancing/balance master/treadmill perturbation training.	**Speed** (immediately) PRT+ Balance+ **balance confidence** (12-month carryover) Balance+

Shen & Mak [52]	45 participants; H&Y 2–3	3–5x/wk; 12 wk	Same PRT as [51].	Active: Technology-assisted balance and gait training.	**Falls** technology-assisted balance and gait training+

ACSM-American College of Sports Medicine. Volume: the total amount of exercise. PRT-Progressive Resistance Training. MFC- Modified Fitness Count. ROM- Range of Motion. QoL-Quality of Life. H- Hypertrophy. F- Functional. FOG- Freezing of Gate. PRTI- Progressive Resistance Training with Instability. TTV- Total Training Volume. S- strength. P- Power. TMR - Traditional Motor Rehabilitation. RCFP- Rehabilitation combining falls prevention Con-concentric. Ecc- Eccentric. WK- Week. N- Number. H&Y- Hoehn & Yahar (stage of disease). PD- Parkinson's disease.

+ effect ± small effect >greater effect - no effect ? not definite

*A proportion of ≥70% designated high adherence, and <70% designated low or uncertain adherence. *Score Key: 2 = High Adherence, 1 = Uncertain Adherence, 0 = No Adherence.

Table S12- Amstar 2 Evaluation of Summary Table

**Author(s) & Year**	Q1	Q2	Q3	Q4	Q5	Q6	Q7	Q8	Q9	Q10	Q11	Q12	Q13	Q14	Q15	Q16
Gamborg et al. [25]	Yes	No	Yes	Partial	Yes	Yes	No	Yes	Yes	No	Yes	Yes	Yes	Yes	No	Yes
Padilha et al. [26]	Yes	Yes	Yes	Yes	Yes	Yes	No	Yes	Yes	No	N/A	N/A	Yes	Yes	N/A	Yes
Saltychev et al. [27]	Yes	Yes	Yes	Partial	Yes	No	No	Yes	Yes	No	Yes	Yes	Yes	Yes	Yes	Yes
Ramazzina et al. [28]	Yes	No	Yes	Partial	Yes	Yes	No	Yes	Yes	No	N/A	N/A	Yes	Yes	N/A	Yes
Tonkin et al. [29]	Yes	Yes	Yes	Partial	Yes	No	No	Yes	Yes	No	Yes	Yes	Yes	Yes	Yes	Yes
Xie et al.[30]	Yes	Yes	Yes	Partial	Yes	Yes	No	Yes	Yes	No	Yes	Yes	Yes	Yes	Yes	Yes
Tambosco et al. [31]	Yes	No	Yes	Partial	No	No	No	Yes	No	No	N/A	N/A	No	Yes	N/A	Yes
Uhrbrand et al. [36]	Yes	No	Yes	Partial	No	No	Partial	Yes	Yes	No	Yes	No	Yes	Yes	No	Yes
Tong et al. [37]	Yes	Yes	Yes	Partial	Yes	Yes	No	Yes	Yes	No	Yes	No	Yes	Yes	Yes	Yes
Mao et al. [35]	Yes	Yes	Yes	Partial	Yes	Yes	No	Yes	Yes	No	Yes	No	Yes	Yes	Yes	Yes
Hvingelby et al. [33]	Yes	Yes	Yes	Partial	Yes	No	No	Yes	Yes	No	Yes	No	Yes	Yes	No	Yes

Braz De Oliveira et al. [34]	Yes	No	Yes	Partial	Yes	Yes	No	Yes	Yes	No	N/A	N/A	Yes	Yes	N/A	Yes
Mendes da Costa et al. [32]	Yes	No	No	Partial	Yes	No	No	Partial	Yes	No	N/A	N/A	Yes	Yes	Yes	Yes
Wang et al. [24]	Yes	Yes	Yes	Partial	Yes	Yes	No	Yes	Yes	No	Yes	Yes	Yes	Yes	Yes	Yes
Zhaoli et al. [22]	Yes	No	Yes	Partial	No	No	No	Partial	Yes	No	Yes	No	Yes	Yes	No	No
Roeder et al. [18]	Yes	No	Yes	Partial	Yes	No	No	Yes	Yes	No	Yes	Yes	Yes	Yes	Yes	Yes
Lamotte et al. [20]	Yes	No	Yes	Partial	No	No	No	Yes	No	No	Yes	No	Yes	Yes	Yes	Yes
Hao et al. [19]	Yes	No	Yes	Partial	Yes	No	No	Yes	Yes	No	Yes	Yes	Yes	Yes	Yes	Yes
Gamborg et al. [23]	Yes	Yes	Yes	Yes	Yes	Yes	No	Yes	Yes	No	Yes	Yes	Yes	Yes	Yes	Yes
Chamberlain-Carter & J. [21]	Yes	No	Yes	Yes	Yes	No	No	Yes	Yes	No	N/A	N/A	Yes	N/A	Yes	Yes
Breinesse & Emerson [17]	Yes	No	Yes	Partial	No	No	No	Yes	Yes	No	N/A	N/A	Yes	Yes	N/A	Yes
Zhang et al. [16]	Yes	Yes	Yes	Partial	Yes	Yes	No	Yes	Yes	No	Yes	No	Yes	Yes	No	Yes
Palheta De Lima et al. [15]	Yes	Yes	Yes	Partial	Yes	No	No	Yes	No	No	N/A	N/A	No	Yes	N/A	Yes

Chung et al. [14]	Yes	No	Yes	Partial	Yes	Yes	No	Yes	Yes	No	Yes	No	Yes	Yes	No	Yes
Zhou et al. [13]	Yes	Yes	Yes	Partial	Yes	Yes	No	Yes	Yes	No	Yes	No	Yes	Yes	Yes	Yes
De Almeida et al. [12]	Yes	Yes	Yes	Partial	Yes	Yes	Yes	Yes	Yes	No	Yes	No	Yes	Yes	No	Yes
Alvarez-Bueno et al. [11]	Yes	Yes	Yes	Partial	Yes	Yes	No	Yes	Yes	No	Yes	Yes	Yes	Yes	Yes	Yes
Song et al. [10]	Yes	Yes	Yes	Partial	Yes	Yes	Yes	Yes	Yes	No	Yes	No	Yes	Yes	Yes	Yes
Yang et al. [9]	Yes	Yes	Yes	Partial	Yes	Yes	No	Yes	Yes	No	Yes	Yes	Yes	Yes	Yes	Yes
Tillman et al. [8]	Yes	No	Yes	Partial	Yes	Yes	No	Yes	Yes	No	Yes	No	Yes	Yes	No	Yes
Li et al. [7]	Yes	No	Yes	Partial	Yes	Yes	No	Yes	Yes	No	Yes	No	Yes	Yes	Yes	Yes
Karpodini et al. [6]	Yes	Yes	Yes	Partial	Yes	Yes	No	Yes	Yes	No	Yes	No	Yes	Yes	Yes	Yes
Yang & Wang [5]	Yes	No	Yes	Partial	Yes	Yes	No	Yes	Yes	No	Yes	No	Yes	Yes	No	Yes
Lima et al. [4]	Yes	Yes	Yes	Partial	Yes	Yes	No	Yes	Yes	No	Yes	No	Yes	Yes	Yes	Yes
Paolucci et al. [3]	Yes	No	Yes	Partial	Yes	Yes	No	Yes	Yes	No	N/A	No	Yes	Yes	N/A	Yes
Ernst et al. [2]	Yes	Yes	Yes	Yes	Yes	Yes	Yes	Yes	Yes	Yes	Yes	Yes	Yes	Yes	Yes	Yes
Gollan et al. [1]	Yes	Yes	Yes	Yes	Yes	Yes	No	Yes	Yes	No	Yes	Yes	Yes	Yes	Yes	Yes

RoB- Risk of Bias, N\A- Not Applicable**.**

Q1: PICO components included? **Q2:** Protocol registration? **Q3:** Study design selection justified? **Q4:** Comprehensive search strategy? **Q5:** Duplicate study selection? **Q6:** Duplicate data extraction? **Q7:** Provided list of excluded studies? **Q8:** Details of included studies? **Q9:** Risk of Bias (RoB) assessment technique? **Q10:** Sources of funding for included studies? **Q11:** Appropriate statistical methods for meta-analysis? **Q12:** Impact of RoB on meta-analysis results? **Q13:** RoB considered in the interpretation of results? **Q14:** Explanation/discussion of heterogeneity? **Q15:** Assessment of publication bias? **Q16:** Conflict of interest reported?
